# Natural allelic variation in the CBF2 transcription factor is a pivotal factor controlling cold resistance in potato

**DOI:** 10.1093/plphys/kiaf428

**Published:** 2025-10-15

**Authors:** Ye Chen, Yufan Chu, Jin Wang, Shengxuan Liu, Yingtao Zuo, Chunguang Yao, Jianke Dong, Qingwei Wang, Tiantian Liu, Wei Tu, Jun Qin, Lin Chen, Botao Song

**Affiliations:** National Key Laboratory for Germplasm Innovation and Utilization of Horticultural Crops, Huazhong Agricultural University, Wuhan, Hubei 430070, China; Hubei Hongshan Laboratory, Huazhong Agricultural University, Wuhan, Hubei 430070, China; Key Laboratory of Potato Biology and Biotechnology, Ministry of Agriculture and Rural Affairs, Huazhong Agricultural University, Wuhan, Hubei 430070, China; National Key Laboratory for Germplasm Innovation and Utilization of Horticultural Crops, Huazhong Agricultural University, Wuhan, Hubei 430070, China; National Key Laboratory for Germplasm Innovation and Utilization of Horticultural Crops, Huazhong Agricultural University, Wuhan, Hubei 430070, China; Hubei Hongshan Laboratory, Huazhong Agricultural University, Wuhan, Hubei 430070, China; Key Laboratory of Potato Biology and Biotechnology, Ministry of Agriculture and Rural Affairs, Huazhong Agricultural University, Wuhan, Hubei 430070, China; National Key Laboratory for Germplasm Innovation and Utilization of Horticultural Crops, Huazhong Agricultural University, Wuhan, Hubei 430070, China; Hubei Hongshan Laboratory, Huazhong Agricultural University, Wuhan, Hubei 430070, China; Key Laboratory of Potato Biology and Biotechnology, Ministry of Agriculture and Rural Affairs, Huazhong Agricultural University, Wuhan, Hubei 430070, China; National Key Laboratory for Germplasm Innovation and Utilization of Horticultural Crops, Huazhong Agricultural University, Wuhan, Hubei 430070, China; Hubei Hongshan Laboratory, Huazhong Agricultural University, Wuhan, Hubei 430070, China; Key Laboratory of Potato Biology and Biotechnology, Ministry of Agriculture and Rural Affairs, Huazhong Agricultural University, Wuhan, Hubei 430070, China; Industrial Crop Research Institute, Yunnan Academy of Agricultural Science, Kunming, Yunnan 650502, China; National Key Laboratory for Germplasm Innovation and Utilization of Horticultural Crops, Huazhong Agricultural University, Wuhan, Hubei 430070, China; Hubei Hongshan Laboratory, Huazhong Agricultural University, Wuhan, Hubei 430070, China; Key Laboratory of Potato Biology and Biotechnology, Ministry of Agriculture and Rural Affairs, Huazhong Agricultural University, Wuhan, Hubei 430070, China; National Key Laboratory for Germplasm Innovation and Utilization of Horticultural Crops, Huazhong Agricultural University, Wuhan, Hubei 430070, China; Hubei Hongshan Laboratory, Huazhong Agricultural University, Wuhan, Hubei 430070, China; Key Laboratory of Potato Biology and Biotechnology, Ministry of Agriculture and Rural Affairs, Huazhong Agricultural University, Wuhan, Hubei 430070, China; National Key Laboratory for Germplasm Innovation and Utilization of Horticultural Crops, Huazhong Agricultural University, Wuhan, Hubei 430070, China; Hubei Hongshan Laboratory, Huazhong Agricultural University, Wuhan, Hubei 430070, China; Key Laboratory of Potato Biology and Biotechnology, Ministry of Agriculture and Rural Affairs, Huazhong Agricultural University, Wuhan, Hubei 430070, China; College of Biology and Agricultural Resources, Huanggang Normal University, Huanggang 438000, China; National Key Laboratory for Germplasm Innovation and Utilization of Horticultural Crops, Huazhong Agricultural University, Wuhan, Hubei 430070, China; Hubei Hongshan Laboratory, Huazhong Agricultural University, Wuhan, Hubei 430070, China; Key Laboratory of Potato Biology and Biotechnology, Ministry of Agriculture and Rural Affairs, Huazhong Agricultural University, Wuhan, Hubei 430070, China; Key Laboratory of Biology and Genetic Improvement of Horticultural Crops (South China), Ministry of Agriculture and Rural Affairs, College of Horticulture, South China Agricultural University, Guangzhou 510642, PR China; National Key Laboratory for Germplasm Innovation and Utilization of Horticultural Crops, Huazhong Agricultural University, Wuhan, Hubei 430070, China; Hubei Hongshan Laboratory, Huazhong Agricultural University, Wuhan, Hubei 430070, China; Key Laboratory of Potato Biology and Biotechnology, Ministry of Agriculture and Rural Affairs, Huazhong Agricultural University, Wuhan, Hubei 430070, China

## Abstract

Frost stress poses a serious threat to the potato industry. C-repeat binding factors (CBFs) are key transcription factors involved in plant cold responses and the adaptive evolution of land plants. However, their function and underlying mechanisms in potato remain poorly understood. This study analyzed homologous *CBF2* genes from 46 potato genotypes and revealed significant structural variations, including a critical site (site A) that is closely associated with cold tolerance. There are at least 2 site A types, including the cold-tolerant *Solanum commersonii* type and the cold-sensitive *Solanum tuberosum* type. Overexpression of ScCBF2 significantly enhanced potato cold tolerance, whereas StCBF2 overexpression had a limited effect. We demonstrated that both *ScCBF2* and *StCBF2* improve cold resistance by regulating glutathione *S*-transferase tau (GSTU)- and ZAT10-mediated reactive oxygen species scavenging systems. Notably, ScCBF2 uniquely upregulated *Galactinol synthase 3* (*GolS3*), promoting raffinose biosynthesis. Compared with StCBF2, ScCBF2 exhibited a stronger binding affinity to the *GolS3* promoter, resulting in higher transcriptional activation. Overexpression of *ScGolS3* increased leaf raffinose content and cold tolerance. Furthermore, we confirmed the critical role of site A in the ScCBF2–GolS3 regulatory pathway. In summary, this study highlights the functional divergence caused by structural variations in CBF2, with differential regulation of *GolS3* contributing to cold tolerance. Our work provides insights into the molecular mechanisms underlying cold tolerance in potato and offers potential targets for improving frost resistance in this vital crop.

## Teaser

Structural variation in the potato CBF2 transcription factor boosts cold resistance by modulating raffinose biosynthesis without compromising yield, facilitating cold-resistant breeding.

## Introduction

Low temperature is a major environmental stress that severely affects plant growth and development, thereby restricting their geographical distribution (Zhang et al. 2022; Wu et al. 2024). The C-repeat binding factor (CBF) plays a central role in plant cold tolerance by regulating cold-responsive genes (*COR*) (Park et al. 2015; Zhao et al. 2016; Jia et al. 2016; Song et al. 2021a, 2021b). The evolutionary history of angiosperms has been closely linked to the diversification of the *CBF* gene family. A recent study traced the origin and convergent evolution of *CBF*/*DREB1* genes to the tandem duplication of a DREB III ancestor, resulting in the emergence of 2 CBF/DREB1 prototypes in early angiosperms (Nie et al. 2022). Comparative analyses of *CBF*/*DREB1* genes across 43 species confirmed their exclusive presence in angiosperms and high evolutionary conservation (Li et al. 2020). Moreover, angiosperm responses to global cooling events, such as the Cretaceous–Paleogene (K-Pg) boundary and the Late Cenozoic Ice Ages, involved independent whole-genome duplication events that retained cold-related genes, including *CBF* and *ICE1* (Schulte et al. 2010; Barrero-Gil and Salinas 2017; Shi et al. 2018; Song et al. 2020; Wu et al. 2020; Wang et al. 2022a, 2022b). These findings suggest that *CBF* genes play pivotal roles in the evolution of cold tolerance in angiosperms.

Potato (*Solanum tuberosum* L.) ranks as the fourth most important food crop globally (Liu et al. 2024). However, its susceptibility to cold stress severely restricts its geographic distribution and productivity. Exposure to cold stress during the seedling and tuber developmental stages can cause significant damage, including eye necrosis, leaf desiccation, and stem softening (Chang et al. 2014; Yan et al. 2021). Despite its substantial economic importance, research on the mechanisms underlying cold resistance in potato remains limited, with only a few cold-responsive genes, such as those regulating *CBF* expression, identified to date. For instance, *SaMKK2* enhances cold resistance by transcriptionally regulating *CBF* expression (Chen et al. 2022b), while *SaCBL1* improves both basal and acclimated cold tolerance through *CBF* signaling pathways (Chen et al. 2022a). Notably, both *SaMKK2* and *SaCBL1* were cloned from the freezing-tolerant *Solanum acaule* and successfully overexpressed in the freezing-sensitive *S. tuberosum*. However, whether *CBF* genes derived from different genotypes, such as a freezing-tolerant genotype and a freezing-sensitive genotype, exhibit similar functions in regulating freezing tolerance remains unclear and warrants further investigation.

Structural variations in *CBF* gene sequences have been shown to be related to cold resistance in various plant species. For example, studies in *Arabidopsis* revealed correlations between natural variations in *CBF* gene sequences and freezing tolerance across diverse populations (Hannah et al. 2006; McKhann et al. 2008). Specific nucleotide deletions in *CBF2* were associated with freezing tolerance differences in Arabidopsis populations along the Yangtze River (Kang et al. 2013). In addition, comparative studies of Swedish and Italian Arabidopsis genotypes demonstrated that differences in *CBF2* function contributed to freezing tolerance and adaptive evolution (Gehan et al. 2015; Baker et al. 2022). In potato, a 33-amino acid deletion in the activation domain of *ScCBF1* compared with *StCBF1* was found to influence cold resistance (Li et al. 2018, 2022). Collectively, these findings suggest that structural variations in *CBF* genes are related to plant cold resistance and adaptive evolution. However, how the structural variations in *CBF* genes determine the freezing-tolerance of plants is still unknown.

In this study, we identified sequence variations in CBF2 among diverse potato genotypes and discovered a specific site (site A) that is correlated with cold tolerance. Functional analyses of *ScCBF2* (from the cold-tolerant *Solanum commersonii*) and *StCBF2* (from the cold-sensitive *S. tuberosum*) revealed their differential effects on cold resistance, mediated through transcriptional regulation of *GolS3*, a key gene in raffinose biosynthesis. Furthermore, we employed AlphaFold3 and molecular docking techniques to explore the interactions between CBF2 variants and their target promoters, providing insights into the molecular mechanisms underlying potato cold resistance. These findings lay a theoretical foundation for CBF2 protein engineering and future potato breeding efforts aimed at enhancing cold tolerance.

## Results

### The sequence variation of *CBF2* gene in potato is related to cold resistance

**Figure 1.**
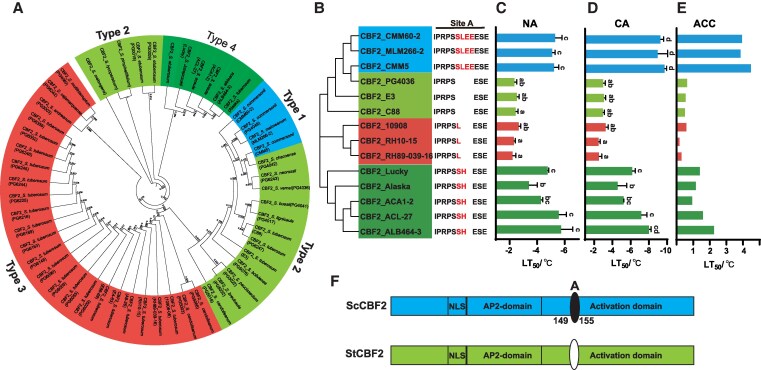
Identification and phylogenetic analysis of ScCBF2 alleles in potato germplasm with differential cold resistance. **A)** Phylogenetic tree of multiple CBF2 homologous protein sequences across the 52 Solanum genus. **B)** Phylogenetic analysis and amino acid presentation of CBF2 site A from 14 potato genotypes with evaluated cold resistance. **C)** and **D)** Semi-lethal temperatures (LT_50_) of these 14 genotypes under NA and CA conditions. NA, noncold acclimation; CA, cold acclimation for 7 d. **E)** Cold acclimation capacity represented by LT_50_ differences between nonacclimated and cold-acclimated states. ACC, acclimation capacity. Letters indicate significant differences among genotypes (*P* < 0.05) with Duncan’s multiple range test. Error bars indicate SD (*n* = 3). **F)** Structural models of ScCBF2 and StCBF2 proteins, highlighting site A distribution, activation domain, nuclear localization signal (NLS), and AP2 domain. The colors in (A, B, F) represent different types of CBF2 sequences, and the same color indicates the same type.

Phylogenetic analysis of *CBF2* homologous genes was conducted using 52 Solanum genus, including 3 *S. etuberosum* accessions, 2 tomato accessions, and 1 *S. melongena* accession, along with 46 *S. tuberosum* interspecies (detailed in Supplementary Table S1). Four amino acid variants at site A were identified across the Solanum genus (Fig. 1A). Phylogenetic tree analysis combined with site A distribution revealed that CBF2 in potato interspecies exhibits a closer evolutionary relationship, aligning with the evolutionary trajectory of potato as described by Tang et al. (2022).

Further analysis integrating phylogenetic relationships with cold tolerance data from 14 potato materials (Fig. 1, B to E, Supplementary Table S2) indicated a strong correlation between site A variations and cold resistance. The serine–proline sequence at site A (Type 4) was directly associated with enhanced direct cold resistance (NA) and cold acclimation (CA) in potato. While the deletion or substitution of site A with leucine (Type 2 or Type 3) was linked to cold sensitivity. For example, CBF2 sequences from an additional 23 cold-sensitive potato accessions belonging to *S. tuberosum* were classified as Type 2 or Type 3. These findings suggest that natural variation at site A plays a critical role in determining the cold acclimation ability of potato.

To further elucidate the effects of structural variations in the CBF2 coding region, we compared 2 potato species with distinct cold acclimation capacities: *S. commersonii* (CMM60-2/CMM5) and *S. tuberosum* (E3). Although ScCBF2 and StCBF2 share high amino acid sequence similarity, variations at site A caused pronounced differences in their predicted spatial protein structures (Fig. 1F, Supplementary Fig. S1, A and B). Interestingly, the expression patterns of *CBF2* during cold stress were similar in both cold-tolerant CMM60-2/CMM5 and cold-sensitive E3 (Supplementary Fig. S1, C and D). These results highlight that the role of site A might be related to the cold resistance of potato. Different site A types derived from cold-tolerant and cold-sensitive genotypes might have different functions in cold tolerance.

### ScCBF2 plays a stronger role in cold tolerance than StCBF2 in potato

To investigate the roles of ScCBF2 and StCBF2 in cold resistance, the full CDS sequences of both genes were cloned into the pBI121 vector, generating transgenic potato lines overexpressing *ScCBF2* or *StCBF2*. To minimize the potential influence of gene dosage on functional analysis, 6 transgenic lines with similar expression levels were selected: OE-*StCBF2*-5, OE-*StCBF2*-8, OE-*StCBF2*-10, OE-*ScCBF2*-21, OE-*ScCBF2*-27, and OE-*ScCBF2*-28 (Supplementary Fig. S2, A and B). Phenotypic observations under greenhouse conditions showed mere dwarfing in all transgenic lines compared with the wild-type (WT) E3 (Supplementary Fig. S2, C and D).

**Figure 2.**
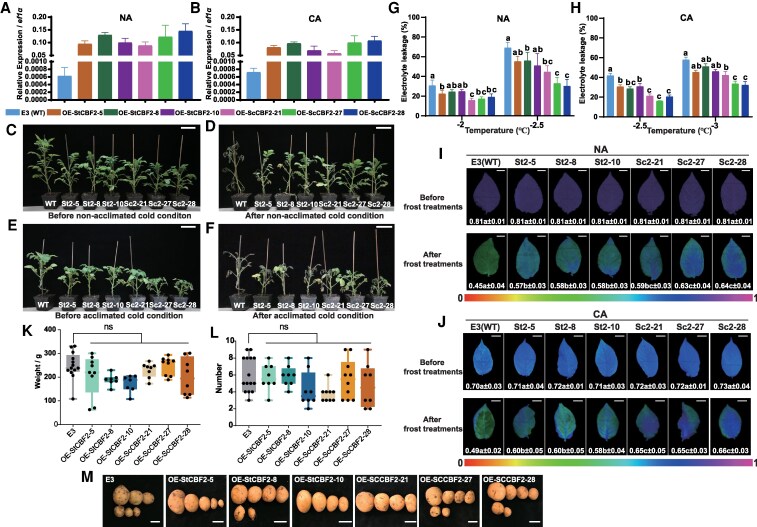
Cold resistance of *ScCBF2* and *StCBF2* overexpression lines under normal and cold-acclimated conditions. **A)** and **B)** Expression levels of *CBF2* in ScCBF2 and StCBF2 transgenic lines compared with the WT E3 under both normal acclimation (NA) and cold acclimation (CA) conditions. Significant differences between the WT and each transgenic line were analyzed using Student's *t*-test (*P < 0.05; **P < 0.01). Error bars represent SD (*n* = 3). **C)** to **F)** Phenotypic comparison of ScCBF2 and StCBF2 transgenic lines and WT E3 before and after frost treatment under NA and CA conditions. Scale bar = 12 cm. **G)** and **H)** EL rates of *ScCBF2* and *StCBF2* transgenic lines and WT E3 after exposure to various temperatures. Significant differences were determined using Duncan’s multiple range test (*P* < 0.05), Error bars indicate SD (*n* = 6). **I)** and **J)** The maximum photochemical efficiency of photosystem II (Fv/Fm) in E3 and *ScCBF2* and *StCBF2* transgenic lines before/after frost treatment under NA and CA conditions. Significant differences were determined using Duncan’s multiple range test (*P* < 0.05), data are presented as means ± SD (*n* = 3). The false color code depicted at the bottom of the images ranges from 0 (red) to 1 (purple). Images were digitally extracted for comparison; the scale bar (1 cm) applies to all images. **K)** and **L)** Tuber weight and number per plantlet across different genotypes. Significant differences were analyzed using a Student’s *t*-test between WT and each transgenic line (*P < 0.05; ns: not significant). Data are presented as means ± SD (*n* = 9). **M)** Representative tuber phenotypes displayed by each genotype. Scale bar = 5 cm.

The expression levels of *CBF2* in transgenic lines and WT E3 were assessed by RT-qPCR under normal conditions and after 7 d of cold acclimation. Although *CBF2* expression levels slightly decreased after cold acclimation for all genotypes, overexpression of *CBF2* was maintained in all transgenic lines under both conditions (Fig. 2, A and B).

To evaluate cold resistance of the lines, 6 transgenic lines and WT E3 plants grown under normal conditions for approximately 35 d were subjected to frost treatments. Without cold acclimation, WT E3 showed more severe frost damage compared with the transgenic lines, with *ScCBF2* transgenic lines exhibiting significantly less damage than *StCBF2* lines (Fig. 2, C and D). After cold acclimation, the same pattern was observed as: *ScCBF2* transgenic lines showed greater resistance to frost, while WT E3 was the most severely damaged (Fig. 2, E and F).

Electrolyte leakage (EL) rates were measured to quantify leaf injury. Consistent with phenotypic observations, WT E3 exhibited significantly higher EL rates than the transgenic lines under both nonacclimated and cold-acclimated conditions. At −2 °C and −2.5 °C without cold acclimation and at −2.5 °C and −3 °C after cold acclimation, *ScCBF2* transgenic lines consistently demonstrated lower EL rates than StCBF2 lines (Fig. 2, G and H). The result of Fv/Fm detection showed the same trend (Fig. 2, I and J). Before exposure to frost treatment, *ScCBF2* and *StCBF2* transgenic lines have similar Fv/Fm values to WT E3 in both noncold acclimation and cold acclimation groups, but all cold-acclimated plants exhibited significantly lower Fv/Fm values compared with nonacclimated plants after the cold acclimation treatment. After exposure to frost treatment, WT E3 had significantly lower Fv/Fm values than all *ScCBF2* and *StCBF2* transgenic lines. It indicated that the transgenic lines overexpressing *CBF2* suffered from less injury compared with WT E3. Overexpression of both *ScCBF2* and *StCBF2* could enhance hardiness. Moreover, *ScCBF2* transgenic lines showed significantly higher Fv/Fm values than *StCBF2* lines.

These results demonstrate that overexpression of *ScCBF2* significantly enhances both constitutive freezing tolerance and cold-acclimated freezing tolerance in potato. In contrast, the effect of *StCBF2* on cold resistance was comparatively weaker, highlighting the functional superiority of *ScCBF2* in conferring cold tolerance.

To comprehensively evaluate the effects of *ScCBF2* and *StCBF2* expression, we extended our analysis beyond plantlet phenotype and cold resistance to examine tuber production. Intriguingly, we observed no significant differences in either tuber weight per plantlet or tuber number per plantlet among WT E3 and *CBF2*-overexpressing transgenic lines (Fig. 2, K and L). This suggests that while *CBF2* overexpression impaired vegetative growth, it did not adversely affect tuber yield. Furthermore, visual examination revealed no apparent differences in tuber morphology between WT and transgenic lines (Fig. 2M).

### ScCBF2 enhances freezing tolerance via increasing the expression of *GolS3* and the content of raffinose

**Figure 3.**
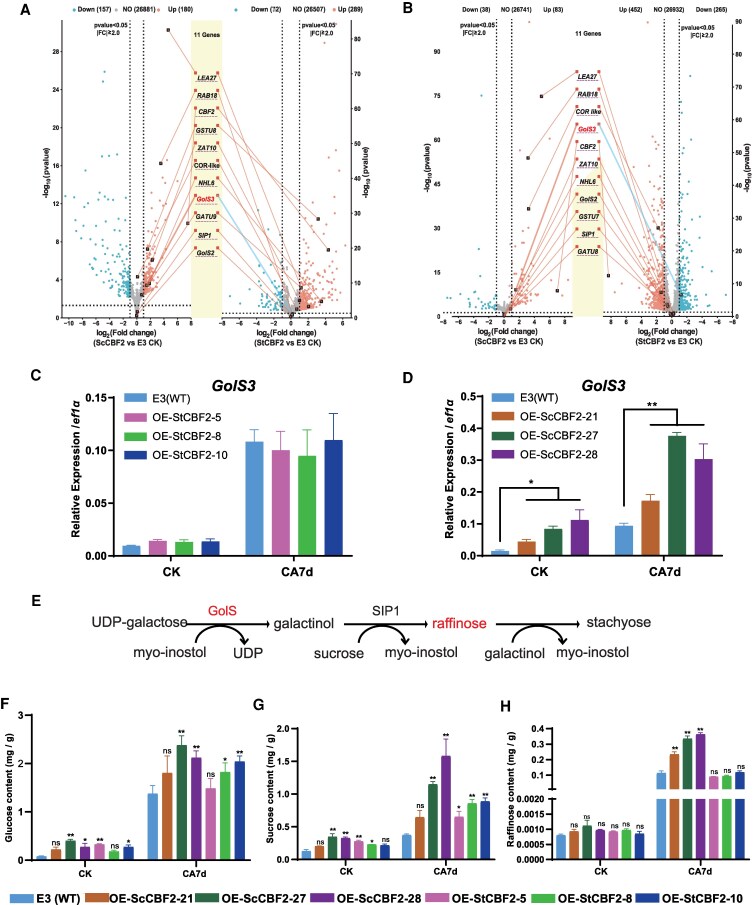
Differences in pathways and genes mediated by *ScCBF2* and *StCBF2*. **A)** and **B)** Double volcano plots displaying common and uniquely regulated genes in *ScCBF2* and *StCBF2* transgenic lines under noncold acclimation (NA) (A) and cold acclimation (CA) conditions (B), based on RNA-Seq data. Blue dots denote down-regulated genes, and red dots represent up-regulated genes. DEGs were defined as those with |fold change| ≥ 2 and a corrected *P* < 0.05. **C)** and **D)** Expression of *GolS3* in *ScCBF2* and *StCBF2* transgenic lines under NA and CA conditions, determined by RT-qPCR. **E)** Schematic of a raffinose biosynthesis pathway. **F)** to **H)** Quantification of glucose (F), sucrose (G), and raffinose (H) contents in *ScCBF2* and *StCBF2* transgenic lines under NA and CA conditions. Significant differences were analyzed using a Student’s *t*-test between WT and each transgenic line (*P < 0.05; **P < 0.01). Error bars indicate SD (*n* = 3).

To identify the functional differences between ScCBF2 and StCBF2, RNA-seq was carried out to analyze differential expression patterns between the *ScCBF2* and *StCBF2* overexpression lines under normal temperature and cold acclimation. At normal temperature, the number of differentially expressed genes (DEGs, fold change ≥2, corrected *P* < 0.05) in OE-*ScCBF2*-27 and OE-*StCBF2*-8 compared with WT E3 was 338 and 362, respectively (Fig. 3A). After cold acclimation, the DEG numbers were 121 and 717, respectively (Fig. 3B).

KEGG enrichment analysis indicated that both *ScCBF2* and *StCBF2* influence the glutathione metabolism pathway (map00480) (Supplementary Fig. S3, A and B). Glutathione *S*-transferase (GST) enzymes (EC: 2.5.1.18), key components of this pathway (Supplementary Fig. S3C), play critical roles in stress responses, including cold and salinity stress. Among the GST genes, *GSTU7* and *GSTU8* were significantly upregulated in both transgenic lines, regardless of cold acclimation (Fig. 3, A and B, Supplementary Fig. S3, D and E). Additionally, genes associated with cold stress responses, such as *COR like*, *Late Embryogenesis Abundant 27* (*LEA27*), and *ZAT10*, were differentially expressed in the transgenic lines, further underscoring their role in cold resistance (Fig. 3, A and B). These findings suggest that both ScCBF2 and StCBF2 enhance cold tolerance in potato by bolstering antioxidant capacity through the glutathione metabolism pathway and cold-responsive proteins.

Further, *GolS3*, a key enzyme involved in raffinose biosynthesis, was uniquely and significantly upregulated by *ScCBF2* at room temperature, with no corresponding change in *StCBF2* transgenic plants. After cold acclimation, *GolS3* expression remained elevated in OE-*ScCBF2* but decreased in OE-*StCBF2* plants compared with WT E3 (Fig. 3, A and B). These findings were validated by RT-qPCR, which confirmed that *ScCBF2* strongly upregulated *GolS3* expression, whereas *StCBF2* exerted only a minimal and transient effect (Fig. 3, C and D).

GolS catalyze the biosynthesis of raffinose oligosaccharides (RFOs), which are crucial for stress tolerance and sugar metabolism (Salvi et al. 2021). In the potato genome, 3 *GolS* genes were identified (He et al. 2023), but only *GolS3* was significantly induced by *ScCBF2*. Neither *GolS1* nor *GolS2* showed notable expression changes in the *ScCBF2* or *StCBF2* transgenic lines under either normal or cold-acclimated conditions (Supplementary Fig. S4, A to D).

The expression pattern of *GolS3* was also detected in both CMM and E3 genotypes. During early cold stress, *GolS3* exhibited co-expression with *CBF2* in both CMM and E3 genotypes, with significantly higher expression in CMM at 4 °C (Supplementary Fig. S4, E and F). The accumulation pattern of raffinose was consistent with *Gols3* expression. The raffinose content showed no significant difference in CMM and E3 at the normal temperature. After exposure to acclimation, CMM showed higher raffinose content than E3 (Supplementary Fig. S4G). Additionally, sucrose and glucose were also detected in CMM and E3, and the result showed that CMM also showed higher sucrose and glucose content than E3 (Supplementary Fig. S4, H and I). The sugar content was further detected in the transgenic lines and WT E3. The content of raffinose was similar in all the transgenic lines and WT E3 at normal temperature. However, after cold acclimation, raffinose content was significantly higher in *ScCBF2* transgenic lines compared with WT, consistent with the *GolS3* expression pattern (Fig. 3, F to H). While the raffinose content of *StCBF2* transgenic lines showed nearly unchanged compared with WT E3. It indicated that ScCBF2, instead of StCBF2, could improve both the expression of *Gols3* and raffinose content after cold acclimation.

The contents of sucrose and glucose were also detected in the transgenic lines and WT E3. Because the 2 types of sugars were significantly improved in CMM, the accumulated trend was consistent with *Gols3*. Gols3 may affect the contents of the 2 sugars. The result showed that the 2 types of sugars showed similar variation in *ScCBF2* and *StCBF2* transgenic lines before/after cold acclimation. It implied that ScCBF2 could affect only raffinose content after cold acclimation.

### ScCBF2 specifically binds to the *GolS3* promoter and activates its transcription in potato

**Figure 4.**
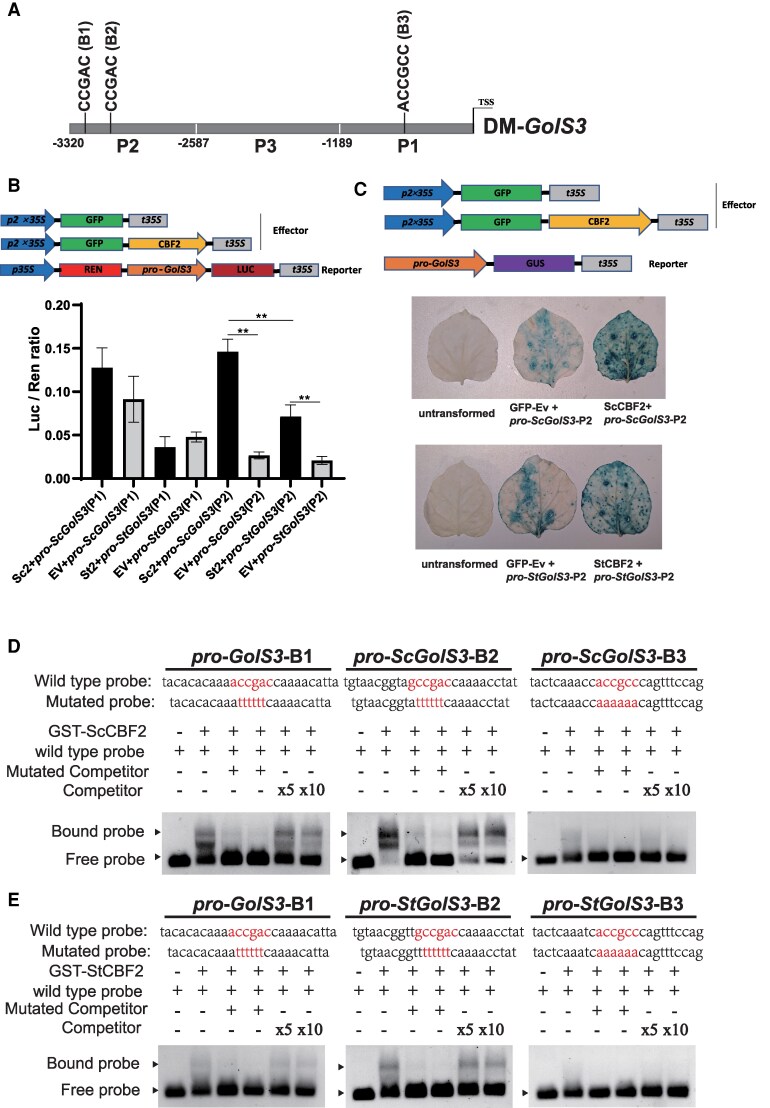
Binding and transcriptional activation of ScCBF2 and StCBF2 on *GolS3* promoters. **A)** Distribution of DRE/CRT elements in the *GolS3* promoter. The promoter was divided into 3 regions (P1, P2, and P3), which contains 3 cis-elements (B1, B2, and B3). **B)** Dual-LUC assay showing the transcriptional activation of ScCBF2 and StCBF2 on P1 and P2 fragments of the *GolS3* promoter. Statistically significant differences between the treatments were determined using a *t*-test. **, *P* < 0.01. Error bars indicate SD (*n* = 4). **C)** Evaluation of ScCBF2 and StCBF2 activity on P2 fragments by GUS transient expression assay in *N. benthamiana*. Scale bar = 1 cm. **D)** and **E)** EMSA demonstrating which DRE/CRT cis-elements (B1, B2, and B3) can be bound by ScCBF2 and StCBF2.

To further explore the regulatory relationship between CBF2 and *GolS3*, we cloned the promoters of the homologous *GolS3* genes, *StGolS3* and *ScGolS3*, from E3 and CMM60-2, respectively, using the reference sequences from DM1-3 (http://spuddb.uga.edu/dm_v6_1_download.shtml). Only a few base pair differences were observed between the promoter sequences of *StGolS3* and *ScGolS3*. Previous studies in model plants, such as *Arabidopsis thaliana*, have shown that CBF proteins can activate downstream gene expression by binding to the core DRE/CRT binding motifs (CCGAC) in the promoters of target genes (Song et al. 2021a, 2021b). In this study, 3 CBF2 potential binding sites (B1, B2, and B3) were identified in the promoter regions of *StGolS3* and *ScGolS3*. All the promoter region was split into 3 parts: P1 (containing CBF2 potential binding site B3), P2 (containing CBF2 potential binding sites B2 and B3), and P3 (Fig. 4A, Supplementary Data S1).

A Dual-Luciferase Reporter (Dual-LUC) assay was conducted to assess whether ScCBF2 and StCBF2 could bind to the P1 and P2 regions of the *GolS3* promoters and activate transcription. The results showed that the co-expression of pro-*ScGolS3*-P2::LUC with 35S::*ScCBF2* exhibited significantly higher luminescence activity than pro-*StGolS3*-P2::LUC with 35S::*StCBF2* (Fig. 4B). Additionally, a GUS reporter assay was performed, and GUS staining was significantly stronger in *Nicotiana benthamiana* leaves containing both pro-*ScGolS3*-P2::LUC and 35S::*ScCBF2*, compared with those containing pro-*StGolS3*-P2::LUC and 35S::*StCBF2* (Fig. 4C).

To further investigate the direct binding of CBF2 to the *GolS3* promoters, we performed an electrophoretic mobility shift assay (EMSA) using purified GST-tagged ScCBF2 and StCBF2 proteins expressed in *Escherichia coli* Rosetta (*DE3*) (Supplementary Fig. S5A). When incubated with biotin-labeled WT probes, protein–DNA complexes were strongly detected for both GST-ScCBF2 and GST-StCBF2 (Fig. 4, D and E, Supplementary Fig. S5B). The addition of an unlabeled competing probe reduced the strength of the protein–DNA complexes in a dose-dependent manner (Fig. 4, D and E, Supplementary Fig. S5B). Moreover, incubation with mutant probes showed no significant effect on the binding capacity of either GST-ScCBF2 or GST-StCBF2, indicating specific binding to the target sites (Fig. 4, D and E, Supplementary Fig. S5B). These results suggest that ScCBF2 can directly and specifically bind to the B1 and B2 sites of the *ScGolS3* promoter (Fig. 4D), while StCBF2 shows weaker binding to the corresponding sites in the StGolS3 promoter (Fig. 4E).

Protein–DNA docking simulations further supported these findings, showing that both ScCBF2 and StCBF2 can bind to the B1 and B2 sites (Supplementary Fig. S5, C and D). Overall, these results indicate that ScCBF2 and StCBF2 can bind to the promoters of *ScGolS3* and *StGolS3*. However, the higher transcriptional activation observed between ScCBF2 and pro-*ScGolS3*-P2 compared with StCBF2 and pro-*StGolS3*-P2 may lead to higher expression of *GolS3* in *ScCBF2* transgenic lines than in *StCBF2* transgenic lines.

### *ScGolS3* positively regulates raffinose synthesis and improves cold resistance in potato

**Figure 5.**
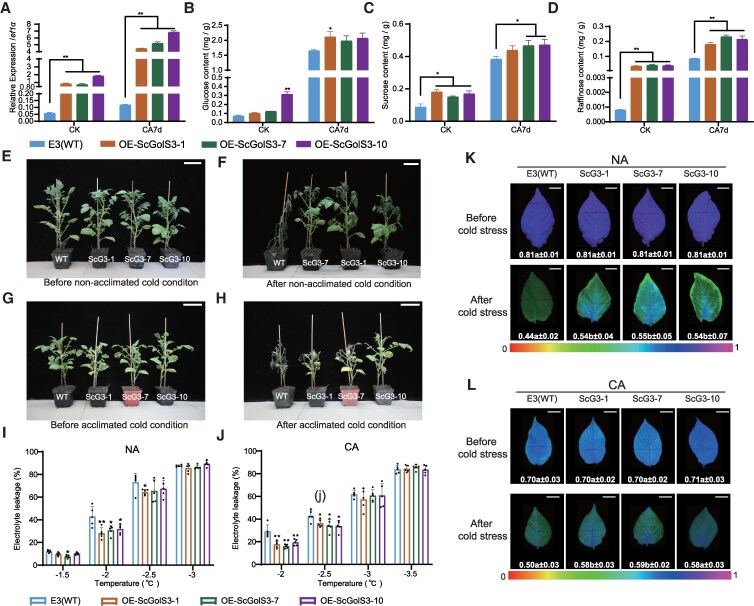
Functional analysis of *ScGolS3* in raffinose biosynthesis and cold resistance. **A)** Expression of *GolS3* in *ScGolS3* transgenic lines and WT E3 under NA and CA conditions. **B)** to **D)** Glucose (B), sucrose (C), and raffinose (D) content in *ScGolS3* transgenic lines and WT E3 under NA and CA conditions. Significant differences were analyzed using a Student’s *t*-test between WT and each transgenic line (*P < 0.05; **P < 0.01). Data are presented as means ± SD (*n* = 3). **E)** to **H)** Phenotypes of *ScGolS3* overexpression transgenic lines and WT E3 before and after frost treatment under NA and CA conditions. Scale bar = 12 cm. **I)** and **J)** EL rates of *ScGolS3* transgenic lines and WT E3 under NA and CA conditions. Significant differences were analyzed between WT E3 and transgenic line using Student's *t*-test (*P < 0.05; **P < 0.01). Error bars indicate SD (*n* = 5). **K)** and **L)** The maximum photochemical efficiency of photosystem II (Fv/Fm) in WT and *ScGolS3* transgenic lines after frost treatment under NA and CA conditions. Significant differences were determined using Duncan’s multiple range test (*P* < 0.05), data are presented as means ± SD (*n* = 3). The false color code depicted at the bottom of the images ranges from 0 (red) to 1 (purple). Images were digitally extracted for comparison; the scale bar (1 cm) applies to all images.

*GolS* genes have been implicated in plant responses to abiotic stress. The ScGolS3 from CMM60-2 and the StGolS3 from E3 have extremely high amino acid sequence similarity and the same domain structure, which indicates that their functions are the same (Supplementary Fig. S6A). To investigate the cold resistance function of *ScGolS3*, it was inserted into the pH7lic vector, with overexpression driven by the 35S promoter in the cold-sensitive material E3. Three transgenic lines (OE-*ScGolS3*-1, OE-*ScGolS3*-7, and OE-*ScGolS3*-10) with strong overexpression of *ScGolS3* were selected based on RT-qPCR results for further cold resistance assessments (Supplementary Fig. S6, B and C). Expression analysis confirmed significant overexpression of *ScGolS3* in these transgenic lines, both at room temperature and after cold acclimation (Fig. 5A).

Subsequently, the content of glucose, sucrose, and raffinose in the transgenic lines and WT E3 was measured at room temperature and after cold acclimation. The results showed that the levels of all 3 sugars were significantly higher in the transgenic potato leaves compared with WT E3 (Fig. 5, B to D).

Under normal conditions, no phenotypic differences were observed between the *ScGolS3* transgenic plants and WT E3 (Fig. 5, E and G). However, after exposure to frost, the transgenic plants exhibited significantly less injury than WT E3, with only minor damage observed in the transgenic lines (Fig. 5, F and H). EL assays further confirmed that the transgenic lines had significantly lower EL compared with WT E3 at both −2 °C and −2.5 °C following cold acclimation (Fig. 5, I and J). The results of Fv/Fm also showed that *ScGolS3* transgenic plants suffered significantly less freezing damage than WT (Fig. 5, K and L).

These results indicate that ScGolS3 enhances both constitutive freezing tolerance and cold resistance after cold acclimation in potato.

### The site A of ScCBF2 positively affects its binding ability to the promoters of *GolS3* in potato

**Figure 6.**
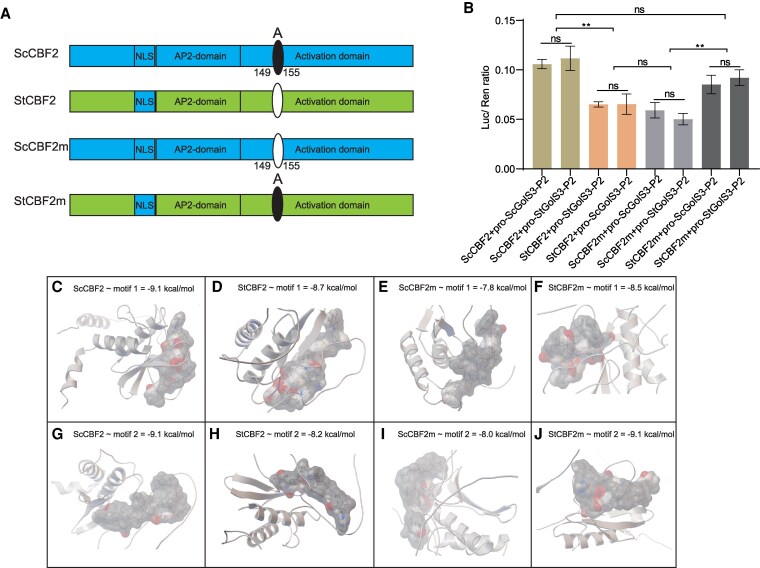
Molecular docking of ScCBF2/StCBF2 and their mutants with GolS3 promoter motifs. **A)** ScCBF2 m represents the ScCBF2 mutant lacking site A, while StCBF2m is the StCBF2 mutant with site A inserted. **B)** Dual-LUC assay evaluating transcriptional activation of ScCBF2, ScCBF2m, StCBF2, and StCBF2m on P2 fragments of *ScGolS3* and *StGolS3*. Significant differences were analyzed using a Student’s *t*-test between WT and each transgenic line (**P < 0.01; ns: not significant). Data are presented as means ± SD (*n* = 3). **C)** to **F)** Molecular binding and affinity energies of ScCBF2, StCBF2, ScCBF2m, and StCBF2m with motif 1 (accgac). **G)** to **J)** Molecular binding and affinity energies of ScCBF2, StCBF2, ScCBF2m, and StCBF2m with motif 2(gccgac). Docking and affinity energy calculations were performed using AutoDock Vina.

To investigate the role of site A in ScCBF2-mediated transcriptional activation of *GolS3* expression, we generated a site A loss-of-function mutant, ScCBF2m, through point mutations in the ScCBF2 background. Additionally, we created a site A gain-of-function mutant, StCBF2m, in the StCBF2 background (Fig. 6A). CBFs typically bind to motifs consisting of the sequence (XccgXc) to exert their function (Song et al. 2021a, 2021b). In this study, the promoters of StGolS3 contain 2 main motifs: motif 1 (accgac) and motif 2 (gccgac).

Better than conventional prediction approaches, AlphaFold3 enables the prediction of protein structures suitable for protein–DNA docking studies, offering valuable insights for our research (Abramson et al. 2024). We use AutoDock Vina for molecular docking; the generation of this model entirely relies on the protein structure of CBF2 predicted by AlphaFold 3 (Supplementary Table S3). Among the 9 generated conformations based on each pair of protein–DNA sequence, the structure exhibiting the lowest binding affinity was selected as the representative model based on its highest thermodynamic probability of representing the biologically relevant binding pose. The root mean square deviation (RMSD) value of 0 further validates this selection, demonstrating complete structural congruence with the reference conformation, thereby confirming its reproducibility across independent docking simulations and eliminating potential artifacts arising from local energy minima. Consequently, our selection criteria incorporated both structural convergence (RMSD = 0) and minimal binding affinity to ensure the identification of the most physiologically plausible docking conformation for subsequent structural and functional analyses.

Molecular docking affinity energy analysis revealed that the binding strength of ScCBF2 to motif 2 was stronger than that of StCBF2. In contrast, the binding strength of ScCBF2m to motif 2 significantly decreased, while the binding strength of StCBF2m to motif 2 increased significantly (Fig. 6, C to J). Dual-LUC assay results further confirmed that ScCBF2 most strongly activated pro-*ScGolS3*-P2 and pro-*StGolS3*-P2, while StCBF2m exhibited a similar level of transcriptional activation on these 2 promoters. In contrast, ScCBF2m, due to the absence of site A, showed markedly reduced activation ability for both promoters, similar to that of StCBF2 (Fig. 6B).

SaCBF2, derived from the cold-resistant wild species *S. acaule* (Fig. 1, B to D), was also studied. We mutated site A of its Type 4 to Type 2 and Type 1, naming the mutants SaCBF2sh and SaCBF2SLEE, respectively (Supplementary Fig. S7A). Molecular docking experiments involving these 3 proteins and motifs 1/2 revealed that deletion of the “SH” amino acids reduced the affinity of SaCBF2sh for motif 2, while replacing them with “SLEE” amino acids resulted in binding similar to that of SaCBF2 (Supplementary Fig. S7, B to G). These results suggest that site A positively influences the binding of ScCBF2 to motifs in the promoters of *GolS3*, thereby regulating the transcriptional activation of pro-*ScGolS3* and pro-*StGolS3*.

**Figure 7.**
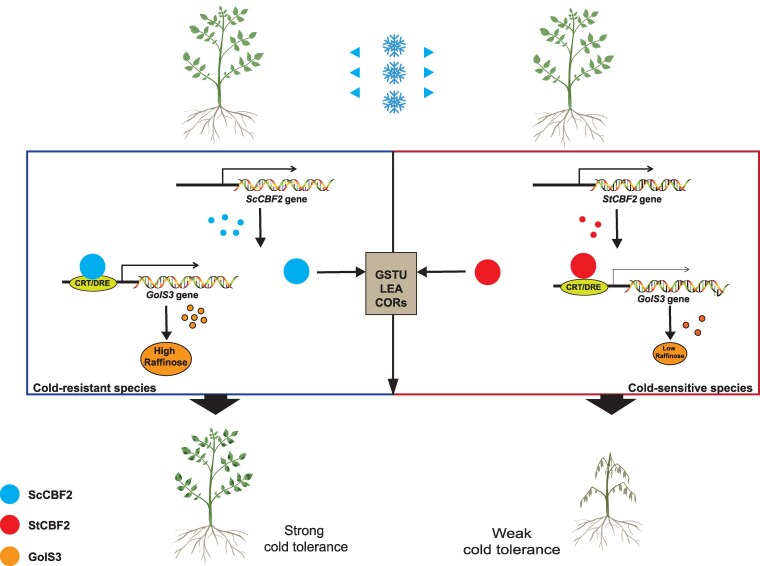
Model depicting the roles of ScCBF2 and StCBF2 in response to cold stress. Blue spots represent the ScCBF2 protein, while red spots indicate the StCBF2 protein, which lacks amino acids at site A relative to ScCBF2. Dark and light yellow spots correspond to ScGolS3 and StGolS3 proteins, respectively. Orange ovals represent raffinose, with the size of each oval proportional to the raffinose content. Solid lines denote established interactions.

Based on these findings, we propose a model explaining the differential roles of ScCBF2 and StCBF2 in regulating cold resistance (Fig. 7). The presence of site A induces structural changes in both ScCBF2 and StCBF2, which affect their transcriptional activation of the *GolS3* promoter. This mechanism results in a significant increase in raffinose content in ScCBF2-overexpressing plants, while raffinose content remains unchanged in StCBF2-overexpressing plants, leading to differences in cold resistance. Additionally, both ScCBF2 and StCBF2 enhance potato cold tolerance by modulating the glutathione transferase and lipid metabolism pathways.

## Discussion

### Deciphering CBF2's functional role in potato is valuable

The C-repeat Binding Factor/Drought Response Element Binding Factor 1 (CBF/DREB1) transcription factors, members of the APETALA2/Ethylene-Response (AP2/ERF) family, are pivotal regulators of plant responses to cold stress (Shi et al. 2018). CBFs exist only in angiosperms and expanded during periods of global cooling geological time, such as the Cretaceous–Paleogene boundary and Late Cenozoic Ice Age (Li et al. 2020; Nie et al. 2022). The emergence of CBFs enhances cold adaptation of angiosperms and might contribute to their wide distribution across the globe (Nie et al. 2022).

In *A. thaliana*, 3 *CBF* genes (*CBF1*, *CBF2*, and *CBF3*) orchestrate cold tolerance (Song et al. 2021a, 2021b). While *CBF1* and *CBF3* have been conclusively identified as positive regulators of cold tolerance, the role of CBF2 remains controversial. For instance, studies on the *cbf2* null mutant reported enhanced cold tolerance (Novillo et al. 2004), suggesting a negative regulatory role. Overexpression of a dominant-negative form of CBF2 led to hypersensitivity to cold stress, supporting its classification as a cold-sensitive gene (Park et al. 2015). However, more recent findings using CRISPR/Cas9-generated mutants indicated that *CBF2* positively regulates cold tolerance and might even play a more significant role than *CBF1* and *CBF3* (Zhao et al. 2016). Given these conflicting reports, clarifying the role of *CBF2* in cold tolerance is of substantial importance.

In this study, we demonstrate that CBF2 positively regulates cold tolerance in potato, providing critical insights into its function in a nonmodel plant species. Recent investigations into the cold stress response pathways in potato have highlighted roles for genes such as *CBL1-like* (*calcineurin B-like 1*) and *MKK2* (*mitogen-activated protein kinase kinase 2*), both of which enhance cold tolerance by upregulating *CBF* expression, including *CBF2* (Chen et al. 2022a, 2022b). However, direct evidence for CBF2's role in potato cold tolerance has been scarce. Through transgenic assays, we confirmed CBF2's freezing-tolerant function, offering a foundation for further exploration of upstream signaling pathways in potato cold stress responses. This report identifies CBF2's freezing-tolerant functional role in potato.

The constitutive overexpression of *CBF* genes in *Arabidopsis* and rice has been shown to enhance freezing tolerance while concomitantly causing growth retardation and yield penalties, thereby limiting their utility in genetic improvement programs targeting freezing stress (Ito et al. 2006; Park et al. 2015; Wei et al. 2022). In our study, while CBF2 overexpression in potato similarly enhanced cold hardiness and induced growth retardation—consistent with observations in *Arabidopsis*—we made the intriguing discovery that it did not negatively impact yield traits or tuber phenotype (Fig. 2). This contrasts with previous findings demonstrating that CBF1 overexpression in potato improved cold tolerance at the expense of yield (Li et al. 2022). Notably, potato CBF2 appears to overcome the typical trade-off between yield and freezing tolerance, making it particularly valuable for breeding programs aimed at enhancing cold tolerance.

### Divergent regulatory mechanisms between *Arabidopsis* and potato CBF2

Our investigation revealed fundamental differences in how CBF2 functions in potato compared with its Arabidopsis homolog. In *A. thaliana*, AtCBF2 acts as a negative regulator of *AtCBF1* and *AtCBF3* expression (Novillo et al. 2007). However, in potato, neither ScCBF2 nor StCBF2 influenced the expression of *CBF1* or *CBF3* (Supplementary Table S4), indicating distinct regulatory mechanisms. These findings suggest that while maintaining similar cold-responsive roles, StCBF2 and AtCBF2 have evolved different regulatory patterns, likely contributing to the extensive evolutionary divergence (approximately 125 million years) between potato and *Arabidopsis* (Kumar et al. 2017).

CBF/DREB1 genes are phylogenetically conserved across angiosperms and were initially presumed to perform similar functions across species (Shi et al. 2018). However, recent research has revealed significant functional divergence through evolutionary processes (Wang et al. 2025). The *CBF* genes originated from tandem duplication of a dehydration-responsive element binding protein (DREB) III gene, with subsequent ε whole-genome duplication generating 2 archetypes (Clades I and II) in ancient angiosperms. Clade II specifically evolved cold-responsive characteristics (Wang et al. 2025). Following divergence of *Arabidopsis* and potato during the Lower Cretaceous (when global temperatures exceeded 18 °C; Wang et al. 2025), Clade II of *Arabidopsis* and potato underwent independent but convergent evolutionary processes, including whole-genome duplications and tandem duplications that ultimately produced CBF2 (Nie et al. 2022). These evolutionary events, coupled with polyploidization, diploidization, and natural selection, have significantly rewired cold-responsive regulatory networks (Wang et al. 2025), which might lead to the distinct regulatory mechanisms of StCBF2 and AtCBF2.

### The different mechanisms of potato ScCBF2 and StCBF2

Under normal growth conditions, both cold-resistant and cold-sensitive potato genotypes maintained comparably low raffinose levels. Following cold treatment, we observed upregulated expression of both *ScCBF2* and *StCBF2*, which similarly activated *glutathione S-transferase* (*GSTU*) and *ZAT10* expression (Fig. 3, A and B). However, a critical functional divergence emerged in their regulation of *GolS3*—while ScCBF2 strongly induced *GolS3* expression (correlating with elevated raffinose content and enhanced freezing resistance in *S. commersonii*), StCBF2 exhibited no such regulatory capacity (Fig. 3). These findings demonstrate that while both proteins share conserved functions in *GSTU/ZAT10* activation, their differential regulation of *GolS3* primarily accounts for their distinct cold resistance phenotypes.

GSTU plays a pivotal role in glutathione metabolism, protecting cellular membranes from oxidative damage during cold stress by degrading lipid peroxides (Dixon et al. 2010; Fig. 3, C to E). This protective mechanism, along with ZAT10-mediated regulation of oxidative stress responses (Liu et al. 2023), likely contributes to the improved cold tolerance observed in both ScCBF2 and StCBF2 transgenic lines.

Our results reveal a striking difference in how ScCBF2 and StCBF2 regulate *galactinol synthase 3* (*GolS3*), a key enzyme in raffinose biosynthesis (Supplementary Fig. S4). Notably, while previous studies reported cold-induced upregulation of *ScGolS1* (He et al. 2023), we observed no such activation of *GolS1* in our transgenic lines (Supplementary Fig. S4, A and C). Complementary experiments demonstrated that *ScGolS3* overexpression in cold-sensitive E3 plants significantly enhanced freezing resistance while increasing raffinose accumulation (Fig. 5), establishing the ScCBF2–GolS3 module as a crucial component for cold adaptation.

The distinct regulatory capacities of ScCBF2 and StCBF2 originate from structural variations in their activation domains (Fig. 6). Structural variations in CBF proteins have been implicated in plants’ temperature-adaptive evolution (Hannah et al. 2006; McKhann et al. 2008). The previously reported structural variations include: (1) Amino acid variation in the AP2/ERF domain causes binding preference. For example, AtCBFs preferentially bind to 5′-A/GCCGAC-3′ sequence (Song et al. 2021a, 2021b), while rice DREB1C specifically binds to 5′-GCCGAC-3′ sequence (Wei et al. 2022; Deng et al. 2024). These different binding preferences enable rice DREB1C to perform distinctive functions, including elevating nitrogen use efficiency, photosynthesis, and grain yield. The R12 residue in OsDREB1C AP2/ERF determines this binding preference (Deng et al. 2024). (2) Single-base insertions or deletions in the transactivation domain of CBF2 cause frameshift mutations and the loss of transactivation activity. These variations in CBF2 contribute to the adaptation of *A. thaliana* populations along the Yangtze River to different January average temperatures (Kang et al. 2013). The similar variations also confer greater freezing sensitivity in Italian genotypes compared with the Swedish ones (Gehan et al. 2015). Our present research provides evidence that amino acid variation in the activation domain can also contribute to the functional divergence of CBF2. Site A could be used as a critical potential target in potato breeding programs aimed at optimizing yield and cold tolerance.

The observed structural differences caused by the absent/present of site A between ScCBF2 and StCBF2 support the hypothesis of rapid evolutionary divergence among CBF transcription factors within the Solanum genus (Carvallo et al. 2011; Aversano et al. 2015). *S. commersonii* (the source of ScCBF2) and *S. tuberosum* (the source of StCBF2) divergent for 2.3-million-year. A 33-amino-acid difference was also present between ScCBF1 and StCBF1 (Li et al. 2018). This rapid evolution may be a generalized phenomenon across the CBF gene family in Solanum.

Overall, this study significantly advances our understanding of CBF2 function in potato, elucidating its unique regulatory pathways and demonstrating its potential for improving cold stress resilience without compromising yield. These findings provide valuable insights for potato breeding programs and contribute to our broader understanding of CBF gene evolution in plants.

## Materials and methods

### Plant materials, growth conditions, and stress treatments

The potato genotypes used in this study included the cold-resistant wild potato species *S. commersonii* accession CMM60-2, CMM5, and the cold-sensitive species *S. tuberosum* accession E3, along with 8 other potato accessions (Akasa Frostness, ALB464-3, ACA1-2, ACL-27, Lucky, MLM266-2, RH89-039-16, 10908-06, C88, RH10-15, PG4036) (Supplementary Table S2). The transgenic potato lines employed in the experiments were: *ScCBF2* overexpression lines (OE-*ScCBF2*-21/27/28), *StCBF2* overexpression lines (OE-*StCBF2*-5/8/10), and ScGolS3 overexpression lines (OE-*ScGolS3*-1/7/10).

Potato plants were grown in 12 cm diameter pots under controlled conditions, with a light/dark cycle of 16 h light/8 h dark and a temperature of 22 °C ± 2 °C for approximately 30 d. One-mo-old plants were used for subsequent experiments. For cold acclimation, plants were transferred to a 4 °C growth chamber under the same light/dark conditions (16 h light/8 h dark) for 7 d. Leaf samples were collected at the appropriate time points for further analysis. For frost treatment, both nonacclimated and cold-acclimated groups were concurrently exposed to differential frost treatments in separate temperature-controlled chambers (−3 °C for nonacclimated and −3.5 °C for cold-acclimated groups). Following a 2-h freezing period, all plants were transferred to complete darkness at ambient temperature for 8 h, then returned to standard growth conditions for 48 h prior to phenotypic assessment through photographic documentation.

### Cold tolerance measurement

The cold tolerance of the various potato materials used in this study, both with and without cold acclimation, was evaluated using the EL rate and Fv/Fm. Leaves from the third, fourth, and fifth compound leaves were collected, and the EL rate was measured following a previously described protocol (Kou et al. 2018; Chen et al. 2022a). Briefly, leaf samples were placed into glass tubes containing 0.5 mL of distilled water and then transferred to a programmable cooling bath (CC-K20, Huber, Berching, Germany) set to 0 °C for 30 min. Distilled water cubes were added to the bottom of each tube to initiate freezing. After an additional 30 min, the temperature was lowered to −1 °C, where it was maintained for 1 h. The temperature was then decreased at a rate of −1 °C per hour, and samples were removed at predetermined temperatures.

After thawing on ice for 8 h, the tubes were equilibrated to room temperature, and 9.5 mL of distilled water was added. The samples were shaken at 160 r/min for 2 h, and the initial electrical conductivity (EC1) was measured at room temperature. The samples were then boiled for 30 min, allowed to return to room temperature, and the final electrical conductivity (EC2) was determined. The relative EL rate was calculated using the formula: (EC1/EC2) × 100%. This method was used to assess the cold tolerance of *ScCBF2*, *StCBF2*, and *ScGolS3* transgenic lines. Additionally, the semi-lethal temperature (LT_50_), representing the freezing tolerance, was calculated based on the EL rate for the 14 potato accessions.

Fv/Fm measurements were carried out as follows: Leaf samples were collected freshly and processed similarly to the EL rate detection protocol, including sampling and gradient cooling in the cooling bath. After gradual cooling to −2.5 °C (for nonacclimated cold tolerance detection) or −3 °C (for acclimated cold tolerance detection) with a 1-h incubation, the leaves were thawed on ice for 8 h. They were then kept in darkness for 30 min before Fv/Fm measurement and imaging using an IMAG-MAXI chlorophyll fluorescence imaging system (Heinz Walz, Effeltrich, Germany).

### RNA extraction and RT-qPCR

Total RNA was extracted from frozen samples using the Plant Total RNA Kit (ZOMANBIO, Beijing, China). First-strand cDNA synthesis was performed with the 5× All-in-One RT Master Mix Reverse Transcription Kit (ABM, Richmond, Canada). Reverse transcription quantitative PCR (RT-qPCR) was conducted using the EvaGreen 2× qPCR Master Mix (ABM) in a LightCycler 480 II (Roche) system. The potato endogenous *ef1α* gene was used as the reference gene. The relative expression of target genes was calculated using the 2^−ΔCt^ method. Primer sequences used for the RT-qPCR assay are provided in Supplementary Table S5. Each treatment was performed with at least 3 biological replicates.

### Vector construction and potato transformation methods

The coding sequences of ScCBF2 (cloned from cold-tolerant CMM60-2) and StCBF2 (cloned from cold-sensitive E3) were subcloned into the pBI121 vector (Xba I and Sac I) via recombination. While *ScGolS3* (cloned from CMM60-2) was subcloned into the pH7lic vector (Stu I). These plasmids, driven by the CaMV 35S promoter, were used for overexpression assays. The constructed plasmids were then transformed into *Agrobacterium tumefaciens* strain GV3101, which was subsequently used to transform the cold-sensitive potato genotype E3 via *Agrobacterium*-mediated transformation as previously described (Si et al. 2003). Positive plantlets were selected on kanamycin-containing medium. The expression of target genes in the transgenic lines and WT E3 was confirmed via RT-qPCR. Among the transgenic lines overexpressing *ScCBF2*, *StCBF2*, and *ScGolS3*, the 3 lines exhibiting the highest overexpression levels were selected for subsequent analysis.

### Agronomic trait measurement

Plantlets were initially cultured on MS medium containing 4% sucrose and 0.7% agar, maintained at 20 ± 1 °C under 60 *μ*mol·m⁻²·s⁻¹ illumination. After 4 wk, plantlets were transplanted into pots for microtuber production. Subsequent tuber yield assessments were conducted on the microtubers using 25 cm diameter pots in greenhouse conditions (15 to 20 °C) at the South Subtropical Crops Research Institute, Zhanjiang, China, from November 20, 2024, to February 19, 2025. For each genotype, tuber weight and number were determined from at least 8 independently potted plantlets.

### RNA-sequencing and analysis

Leaves were collected from 30-d-old plantlets of WT E3, OE-ScCBF2-27, and OE-StCBF2-8, which were grown under normal conditions and after cold acclimation. For each line, 3 independent biological replicates were used. Total RNA was extracted using the Plant Total RNA Kit (ZOMANBIO), and RNA integrity was assessed via 1.5% agarose gel electrophoresis. cDNA libraries were constructed and sequenced using the Illumina HiSeq platform (Berry Genomics). Clean reads were aligned to the *S. tuberosum* genome (version 6.1) from the Potato Genome Sequencing Consortium (http://spuddb.uga.edu/) using the transcript quantification tool Salmon (version 1.4.0). Gene expression levels were quantified by Transcripts Per Million. Differential expression analysis was performed using DESeq2, with DEGs identified based on an absolute log2 fold change ≥ 1 and a false discovery rate < 0.05. Gene Ontology enrichment analysis and heatmaps were generated using TBtools software (Chen et al. 2020).

### Acquisition and phylogenetic tree analysis of *CBF2* homologous genes from multiple potato germplasm

A total of 14 CBF2 homologous genes were cloned from 14 potato accessions, including Akasa Frostness, ALB464-3, ACA1-2, ACL-27, Lucky, MLM266-2, CMM5, CMM60-2, E3, RH89-039-16, PG4036, C88, RH10-15, and 10908-06. The primers used for PCR amplification are listed in Supplementary Table S3. In addition, 24 CBF2 homologous genes were identified through a genome-wide BLAST search across 24 genomic databases, including those of *Solanum melongena*, *Solanum pimpinellifolium*, *Solanum lycopersicum*, and 21 cold-sensitive potato cultivars available at http://solomics.agis.org.cn/potato/species.

Multiple sequence alignment of the *CBF2* homologs was conducted to investigate the conserved regions. The phylogenetic tree was constructed using the Neighbor-Joining method in MEGA X with default parameters. The evolutionary tree was visualized and enhanced using the Interactive Tree of Life tool (https://itol.embl.de/upload.cgi).

### Electrophoretic mobility shift assays (EMSA)

The full coding sequences of *ScCBF2* and *StCBF2* were amplified and cloned into the prokaryotic expression vector pET42b, which includes a GST tag. Recombinant proteins were expressed in *E. coli* Rosetta (DE3) at 16 °C for 14 h, induced by 0.2 mm isopropyl-β-D-thiogalactoside. The GST-ScCBF2 and GST-StCBF2 fusion proteins were purified using Glutathione-Sepharose 4B resin (GE Healthcare). Probes containing CRT/DRE elements were synthesized and labeled with a FAM fluorescent tag, while the core-binding sequence of the mutated probes was replaced with 6 consecutive “T” or “A” nucleotides.

Purified proteins were first incubated with FAM-labeled probes and then subjected to competitive binding assays using both mutated and unmutated probes. Binding interactions were analyzed by agarose gel electrophoresis, and band sizes were determined accordingly. The EMSA was performed as described previously (Liu et al. 2019).

### GUS staining

A histochemical assay was performed to assess β-glucuronidase (GUS) activity through transient expression in *N. benthamiana*. The promoter fragment upstream of the translation start site (TSS) of *GolS3* was amplified from genomic DNA of *S. commersonii* (CMM60-2) and *S. tuberosum* (E3). The cloned promoter fragments were inserted into the pBI121 vector (Hind III and Xba I), containing the GUS gene, to generate pro-ScGolS3-P2-GUS and pro-StGolS3-P2-GUS constructs, respectively. These constructs were transferred into *A. tumefaciens* GV3101 and introduced into the leaves of 6-leaf-stage *N. benthamiana* plants.

Following incubation for 2.5 d under appropriate conditions, leaves co-infiltrated with the genetic constructs were selected for GUS staining and expression analysis. After 2.5 d of normal growth, treated leaves were incubated at 37 °C for 24 h in GUS staining buffer (50 mm sodium phosphate buffer, pH 7.2, 0.1% Triton X-100, 0.1% *N*-laurylsarcosine, 10 mm Na_2_EDTA, 1 mm K_3_Fe(CN)_6_, 1 mm K_4_Fe(CN)_6_, and 0.5 mg/mL 5-bromo-4-chloro-3-indolyl-β-D-glucuronic acid). The stained leaves were then washed in 80% (v/v) ethanol at 80 °C for 30 min to remove chlorophyll.

### Measurement of sugar content

Potato leaves, both before and after cold acclimation, were collected, immediately frozen in liquid nitrogen, and stored at −80 °C for subsequent analysis of sugar and other substances.

For sugar extraction, approximately 50 mg of freshly frozen leaf tissue (twice the sample amount required for raffinose extraction) was transferred to a prechilled 2.0 mL centrifuge tube. The extraction was performed by adding 350 *μ*L of extraction solution (a 3/7 ratio of isopentyl alcohol to methanol, prechilled at 4 °C) to the tissue powder. The mixture was vortexed at room temperature until uniformly dispersed. The tube was then placed in a −20 °C freezer for 2 h, with intermittent shaking 2 to 3 times during the incubation. Afterward, 350 *μ*L of ice-cold water was added, and the mixture was shaken at 4 °C. The mixture was then centrifuged at 13,000 × *g* for 10 min at 4 °C, separating the water-methanol (upper) phase and the chloroform (lower) phase. The water-methanol phase was carefully transferred to a new 1.5 mL centrifuge tube, avoiding any contamination from the chloroform phase or insoluble particles.

Next, 300 *μ*L of ice water was added to the chloroform phase, followed by swirling and centrifugation (under the same conditions as above). The chloroform phase was then transferred to a separate tube. The water-methanol phases from both steps were combined, and approximately 1.0 mL of the extract was evaporated and dried at 35 °C for 3 h in a freeze-concentrating centrifugal dryer to precipitate the dry matter. The precipitates were re-dissolved in 400 *μ*L of water, vortexed, and centrifuged as described earlier. The resulting supernatant was stored at −20 °C or directly processed for analysis.

For analysis, 100 *μ*L of the supernatant was transferred into a new 1.5 mL centrifuge tube for further processing. A second 100 *μ*L sample was also drawn for immediate use in liquid chromatography-mass spectrometry (LC-MS).

Glucose, fructose, and sucrose concentrations were quantified by LC-MS using a reverse-phase Hypersil GOLD column (150 × 2.1 mm, 3 *μ*m, Thermo Fisher Scientific) in an ultra-high performance liquid chromatography system (UltiMate 3000, Thermo Fisher Scientific) set at 70 °C. The mobile phase consisted of 10 mm/L ammonium formate aqueous solution (Phase A) and acetonitrile (Phase B), with a Phase A to Phase B elution ratio of 16:84 and a flow rate of 0.8 mL/min. Each sample was injected at a 1 *μ*L volume. Soluble sugars were detected with a high-resolution mass spectrometer (QExactive Plus, Thermo Fisher Scientific) using the HESI source in negative ion mode. Full MS/dd-MS2 (Top 20) was used for sugar detection, with a resolution of 17,500, an ACG target value of 3e6, and a scanning range of 100 to 450 m/z. For dd-MS2, the resolution was 17,500, with an ACG target value of 1e5, a scan window of ±1.5 m/z, and an impact energy (NCE) of 40.

A standard curve generated from glucose, fructose, and sucrose standards was used to calculate the concentrations of these sugars in the samples, with data analysis performed using XCalibur software. Finally, raffinose, sucrose, and glucose content were normalized to the fresh plant tissue mass, determined by pre-extraction weighing.

### Dual luciferase assay

The upstream fragments of the TSS of *ScGolS3* and *StGolS3* were amplified from the genomic DNA of CMM60-2 and E3 as promoters. The promoter sequences were then cloned into the pGREEN0800II vector (Kpn I and Nco I), which contains both the firefly luciferase (LUC) and sea kidney firefly luciferase (REN) genes, generating the constructs pro-*ScGolS3*-P1/P2-LUC and pro-*StGolS3*-P1/P2-LUC.

The constructed plasmids were transferred into *A. tumefaciens* strain GV3101(with pSoup). The *Agrobacterium* cultures were co-expressed with 35S::ScCBF2, 35S::StCBF2, or an empty vector. Following infiltration into *N. benthamiana* leaves, the plantlets were incubated under appropriate growth conditions for 2.5 d. Specific co-infiltrated leaf combinations were selected for subsequent GUS staining and expression analysis.

After the 2.5-d incubation period, the luciferase activities were assessed using a Dual-Luciferase Reporter Assay Kit (Yeason Biotechnology, Shanghai, China). The activities of LUC and REN were measured, and the LUC/REN ratio was calculated to determine the transcriptional activity of each promoter.

### Molecular docking

The protein spatial structure models of ScCBF2, StCBF2, ScCBF2m, StCBF2m, SaCBF2, SaCBF2sh, and SaCBF2SLEE were generated using AlphaFold3 (https://alphafoldserver.com/about), and the corresponding models were saved in PDB format (Abramson et al. 2024). Additionally, PDB format files for the spatial structure models of motif 1 (accgac) and motif 2 (gccgac) were also acquired.

Molecular docking experiments were performed using AutoDock Vina, with the protein models serving as receptors and the motif models as ligands. The scoring function of AutoDock Vina is a machine learning-optimized semi-empirical potential that integrates weighted combinations of van der Waals forces, hydrogen bonding, electrostatic interactions, and hydrophobic effects, trained on known complex structures to enable rapid prediction of molecular binding affinities. The docking results, including interaction model files and affinity energy values, rmsd l.b. and rmsd u.b., were obtained from the scoring table of the prediction results. Detailed procedural steps for AutoDock Vina were followed as outlined in the literature (Trott and Olson 2010; Eberhardt et al. 2021). A total of 9 models of docking will be generated. We considered both “RMSD = 0” and the lowest binding affinity to identify the optimal docking model for subsequent analysis (Eberhardt et al. 2021).

For visualization and aesthetic image processing of the receptor–ligand interaction models, the receptor files and interaction models were imported into PyMOL software (DeLano 2002).

### Statistical analysis

Experimental data were organized using Microsoft Excel (version 2022). Heatmap generation was performed using TBtools, a bioinformatics software. Statistical analysis was conducted using Student's *t*-tests or 1-way analysis of variance as appropriate. Data are presented as means ± standard deviations (SD), with significant differences between means indicated by asterisks (*, *P* < 0.05; **, *P* < 0.01).

### Accession numbers

Sequence data from this article can be found in the Potato Genome Sequencing Consortium (http://spuddb.uga.edu/) data libraries under accession numbers: *CBF2* (Soltu.DM.03G016730.1); *GolS3* (Soltu.DM.02G024820.1): *GolS1* (Soltu.DM.01G040570.1); *GolS2* (Soltu.DM.01G025230.1): *ZAT10* (Soltu.DM.04G032780.1); *GSTU7* (Soltu.DM.01G029090.1); *GSTU8* (Soltu.DM.09G001320.1); and LEA27(Soltu.DM.01G034240.1).

## Supplementary Material

kiaf428_Supplementary_Data

## Data Availability

The data underlying this article are available in the article and in its online supplementary material.

## Supplementary data

The following materials are available in the online version of this article.

**Supplementary Figure S1.** ScCBF2 and StCBF2 amino acid sequence alignment and the cold-responsive expression of *CBF2* in potato.

**Supplementary Figure S2.** Detection of *ScCBF2*-overexpressing and *StCBF2*-overexpressing transgenic lines.

**Supplementary Figure S3.** The regulatory pathways mediated by ScCBF2 and StCBF2.

**Supplementary Figure S4.** The cold-responsive expression of *GolS3* and the changes in raffinose content in CMM5 and E3.

**Supplementary Figure S5.** ScCBF2 binds to the *StGolS3* promoter.

**Supplementary Figure S6.** The ScGolS3 amino acid sequence and the identification of *ScGolS3* transgenic lines.

**Supplementary Figure S7.** Affinity analysis of SaCBF2 and its mutant variants for motif1/2.

**Supplementary Table S1.** Amino acid types at site A in the CBF2 homologs of *S. melongena*, 2 tomato cultivars, and 32 potato germplasms.

**Supplementary Table S2.** The cold resistance of 14 types of potato materials.

**Supplementary Table S3.** Information on multiple molecular docking models.

**Supplementary Table S4.** DEGs in *ScCBF2*- and *StCBF2*-overexpressing lines and WT E3 with or without cold acclimation.

**Supplementary Table S5.** Primers used in this research.

## Dive Curated Terms

The following phenotypic, genotypic, and functional terms are of significance to the work described in this paper:

ICE1 Gramene: AT3G26744
ICE1 Araport: AT3G26744

MKK2 Gramene: AT4G29810
MKK2 Araport: AT4G29810
