# Supplementary material for: Natural allelic variation in the CBF2 transcription factor is a pivotal factor controlling cold resistance in potato
Source: Plant Physiol. 2025 Oct 15;199(2):kiaf428. doi: 10.1093/plphys/kiaf428 (PMC12559889; doi:10.1093/plphys/kiaf428)
Supplement: kiaf428_Supplementary_Data [file kiaf428_supplementary_data.zip › Supplemental material.pdf]

Supplementary figures:

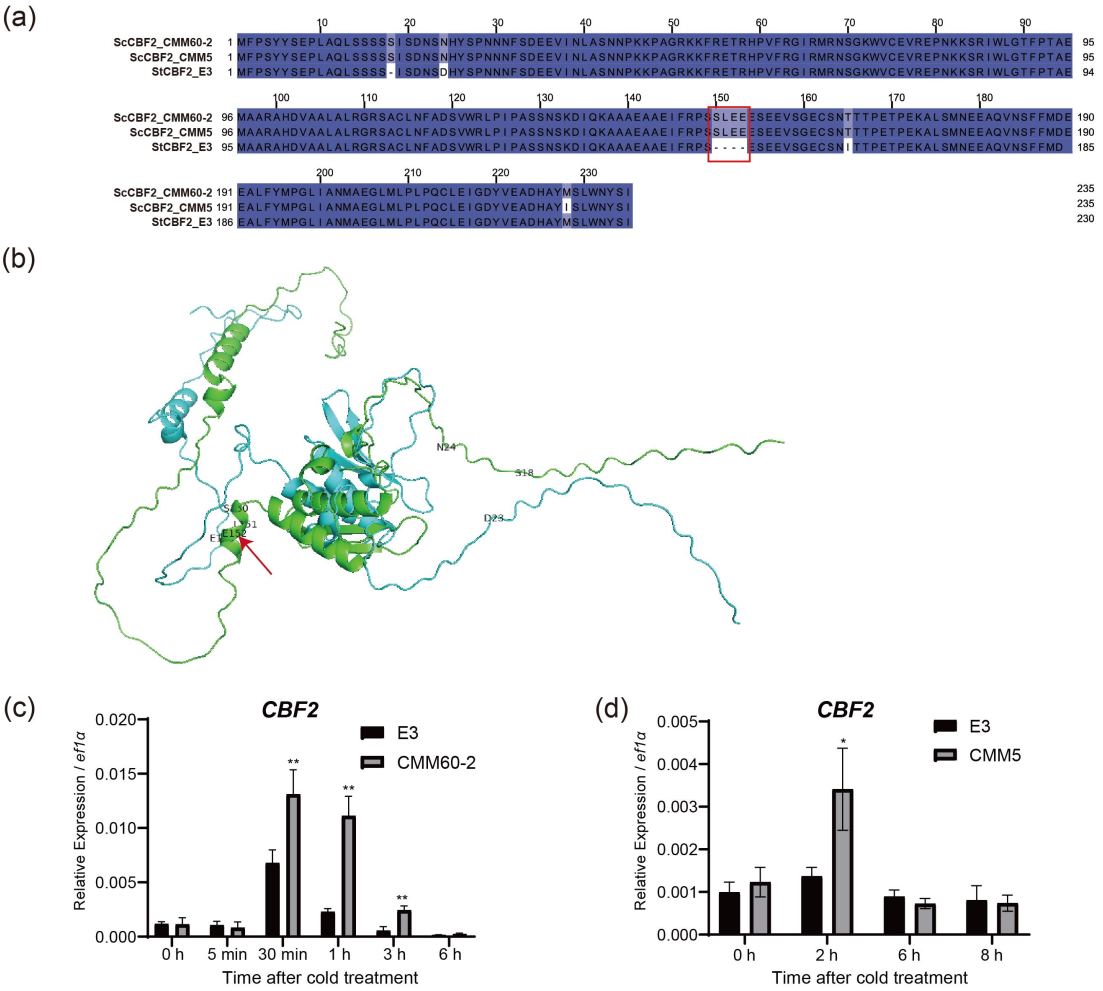

Supplementary Figure S1. ScCBF2 and StCBF2 amino acid sequence alignment and the cold-responsive expression of *CBF2* in potato.

(a) Sequence alignment of three homologous CBF2 amino acid sequences. ScCBF2_CMM60-2 cloned from CMM60-2, ScCBF2_CMM5 cloned from CMM5, and StCBF2_E3 cloned from E3. The red box represents site A of differences. (b) The spatial structural model of ScCBF2 and StCBF2 were predicted using AlphaFold 3 and visualized using Pymol, where the green protein represents ScCBF2, the blue protein represents StCBF2, and the red arrow shows the differential structure of ScCBF2 and StCBF2 (Ser150-Lue151-Glu152-Glu153). N24 stands for Asp, S18 for Ser18, and D23 for Cys23. (c, d) The cold induction expression pattern of the *CBF2* gene in CMM60-2, CMM5 and E3. CMM60-2 and CMM5 are cold-resistant materials, while E3 is a cold-sensitive material. Statistically significant differences were determined using one-way ANOVA. Different letters indicate statistically significant differences at $P < 0.05$. The error bars represent SDs (n = 3).

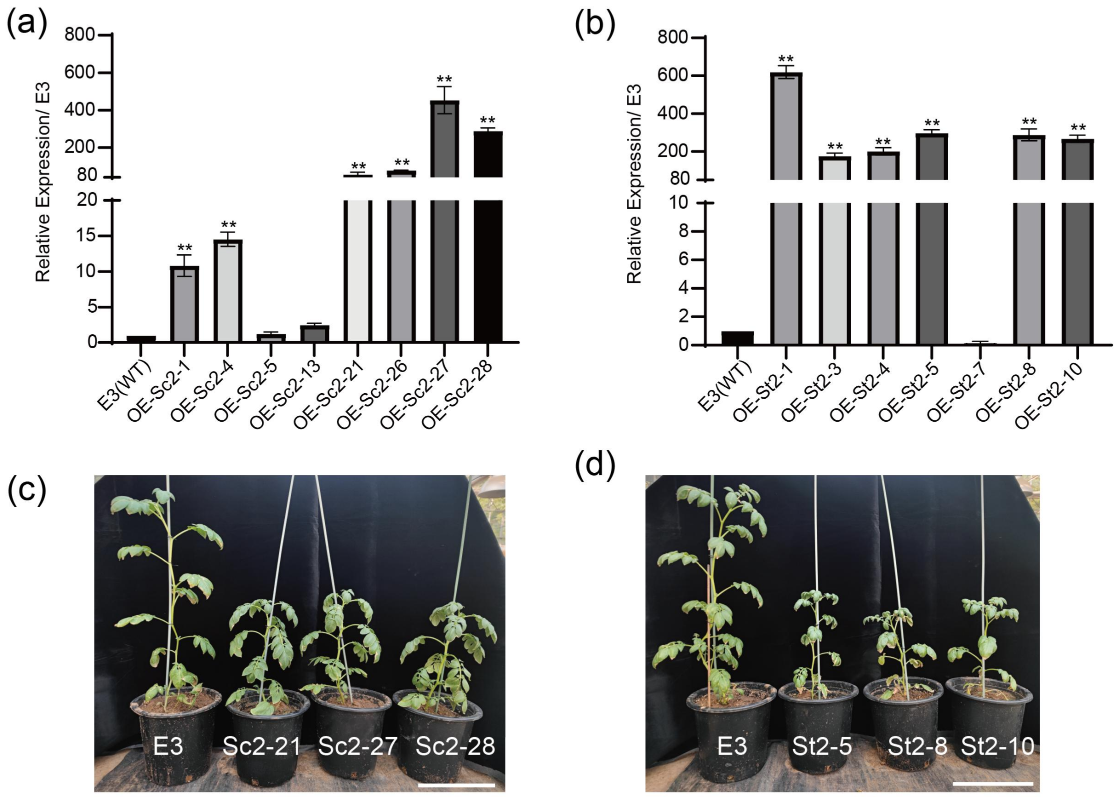

Supplementary Figure S2. Detection of *ScCBF2*-overexprssing and *StCBF2*-overexpressing transgenic lines.

(a, b) The expression of *CBF2* in *ScCBF2* and *StCBF2* transgenic lines by RT-qPCR. (c, d) Plant phenotype of *ScCBF2* and *StCBF2* transgenic plants and wild-type E3 in greenhouse. The scale is 20 cm. The error bars represent SDs (n = 3). Statistically significant differences were determined using *t*-test. **, $P < 0.01$.

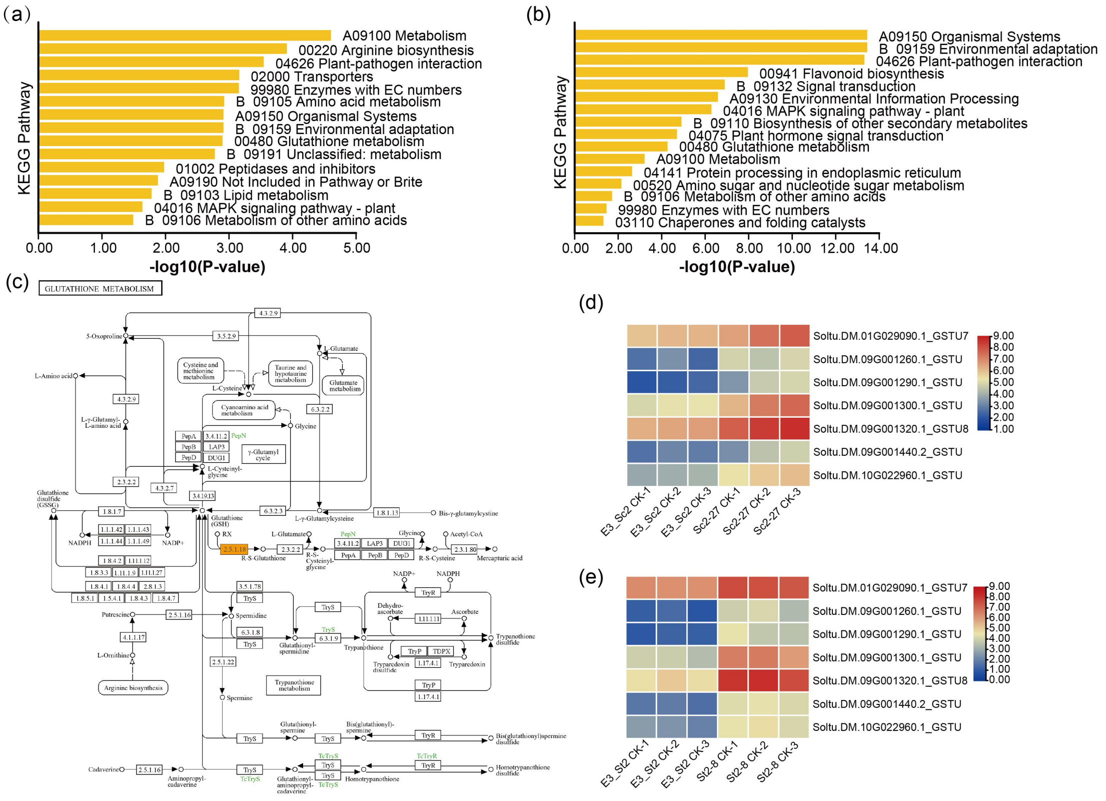

Supplementary Figure S3. The regulatory pathways mediated by ScCBF2 and StCBF2.

(a) The KEGG enrichment results for DEGs between *ScCBF2*-overexpressing lines and the wild-type E3 under normal temperature conditions. (b) The KEGG enrichment results for DEGs between *StCBF2*-overexpressing lines and the wild-type E3 under normal temperature conditions. (c) The Glutathione S-transferase metabolic pathway (map00480). The yellow block represents the upregulated enzyme. The EC number of this enzyme is 2.5.1.18 (brown label), which stands for the GST enzyme. These green letters are important enzymes to this biosynthesis and metabolic networks. (d) and (e) represent the expression levels of some *GSTU* genes in the *ScCBF2* and *StCBF2* overexpressing transgenic lines, respectively. The false color code depicted at the bottom of the images ranges from 1.00 (blue) to 9.00 (red), the value is equal to $\log_2$ (TPM).

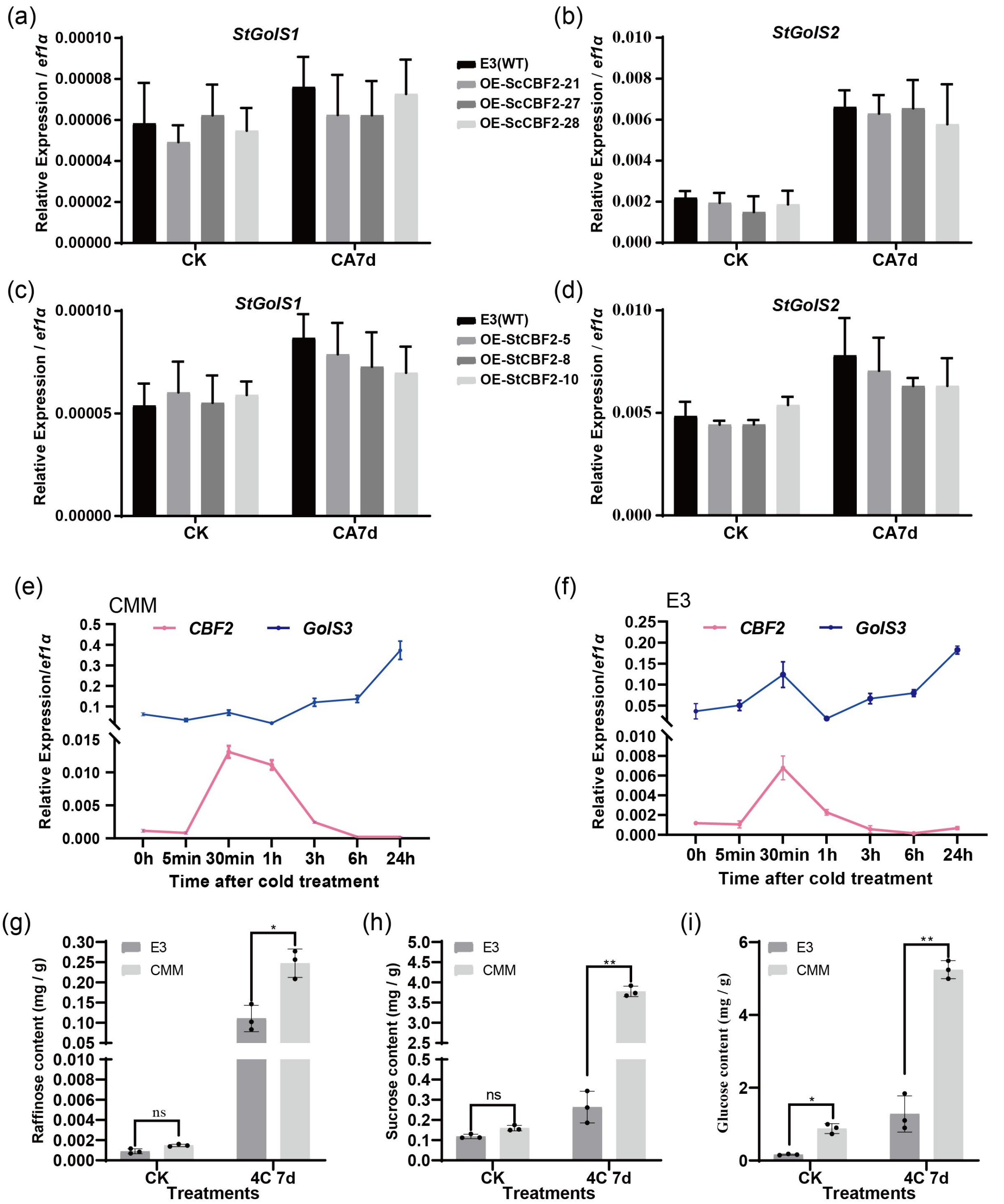

Supplementary Figure S4. The cold-responsive expression of *GolS3* and the changes in raffinose content in CMM5 and E3

(a, b, c, d) The expression of *StGolS1* and *StGolS2* in *ScCBF2* and *StCBF2* transgenic lings. (e, f) The cold induction expression of *GolS3* in CMM5 and E3. (g, h, i) The contents of raffinose, sucrose and glucose in CMM5 and E3 at normal temperature and after cold acclimation for 7 days. All the statistical analyses in this graph were conducted using using *t*-test. *, $P < 0.05$, **, $P < 0.01$. The error bars represent SD (n = 3).

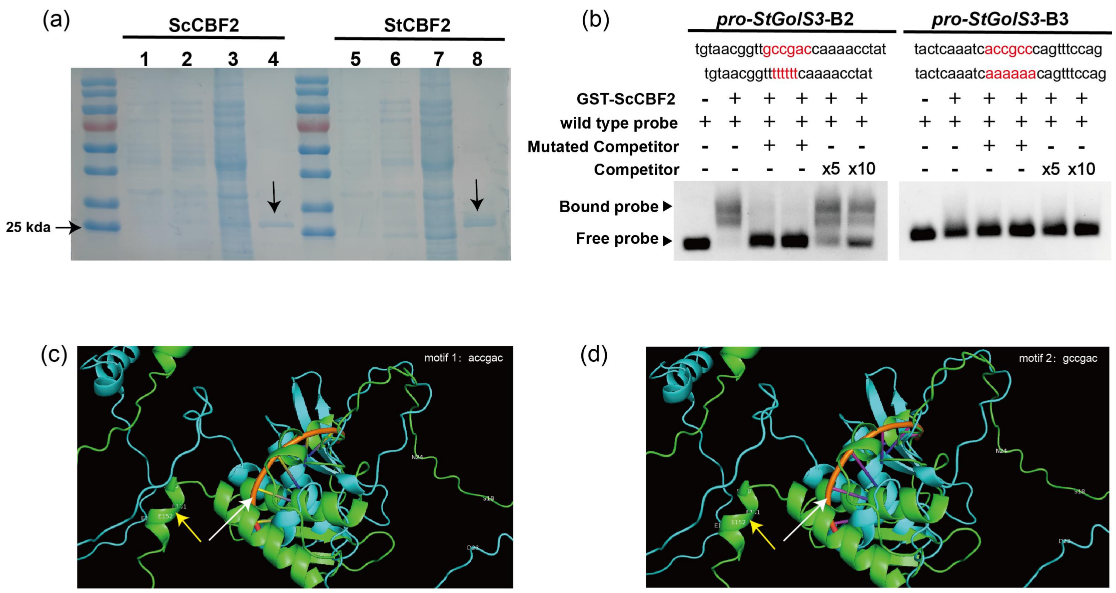

Supplementary Figure S5. ScCBF2 binds to the *StGolS3* promoter.

(a) Purified protein detection of GST-ScCBF2 and GST-StCBF2 showed that lane 1 and 5 were common *Escherichia coli Rosetta* extractions, lane 2 and 6 were non-IPTG-induced extractions from *Escherichia coli Rossett*, lane 3 and 7 were extractions from IPTG-induced expression protein without breaking cells, and lane 4 and 8 were extractions from IPTG-induced expression with cell broken. Protein was purified by GST nickel column filtration. (b) The binding of ScCBF2 to *pro-StGolS3*-B2/B3 was demonstrated by EMSA assay. (c, d) Molecular docking results of ScCBF2 and StCBF2 with motif 1 and motif 2 were visualized using Pymol, wherein the green protein represents ScCBF2, the blue protein represents StCBF2, and the white arrow indicates the spatial structural model of motif 1 and motif 2. The yellow arrow points to site A (Ser150-Lue151-Glu152-Glu153).

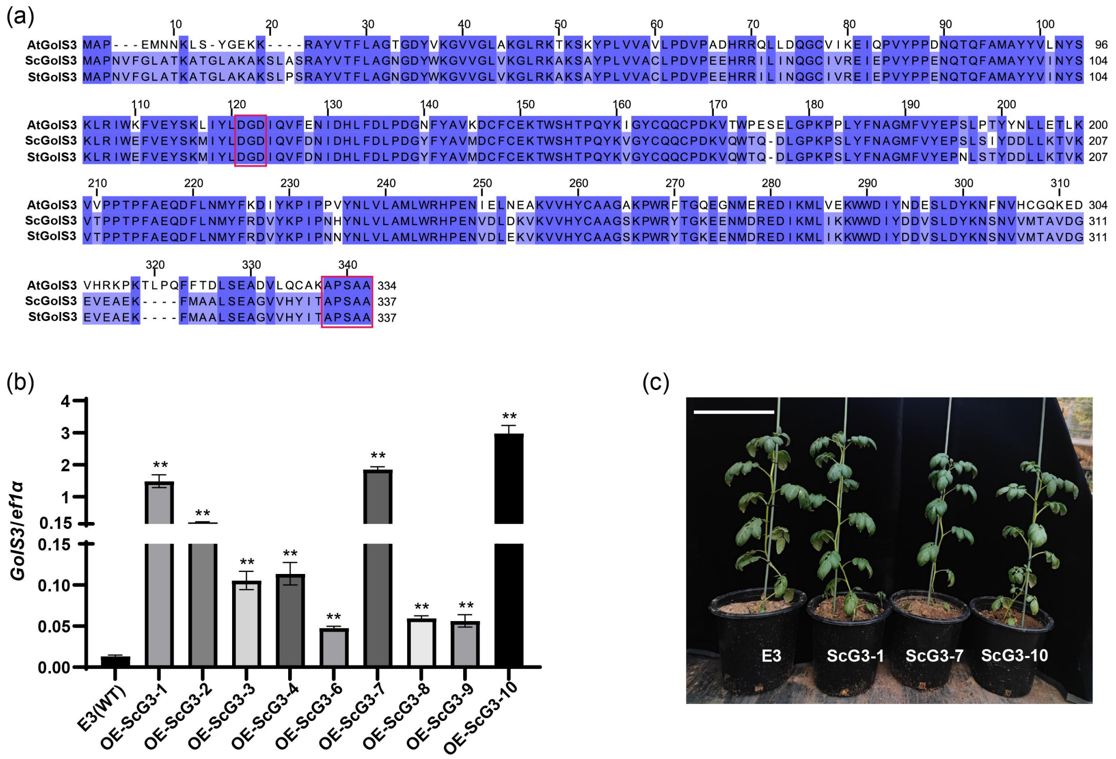

Supplementary Figure S6. The ScGolS3 amino acid sequence and the identification of *ScGolS3* transgenic lines.

(a) Multiple Sequence alignment of amino acid in ScGolS3, StGolS3 and AtGolS3, red boxes being DGD and APSAA domains. *ScGolS3* was cloned from CMM60-2 (*S. commersonii*), *StGolS3* was cloned from E3 (*S. tuberosum*), and *AtGolS3* is *GolS3* in *Arabidopsis thaliana*. (b) The detection of *GolS3* expression of *ScGolS3* transgenic lines and wild-type E3. (c) Plant phenotype of *ScGolS3* transgenic plants and wild-type E3 in greenhouse. The scale is 20 cm. Statistically significant differences were determined using *t*-test. **, $P < 0.01$. The error bars represent SDs (n = 3).

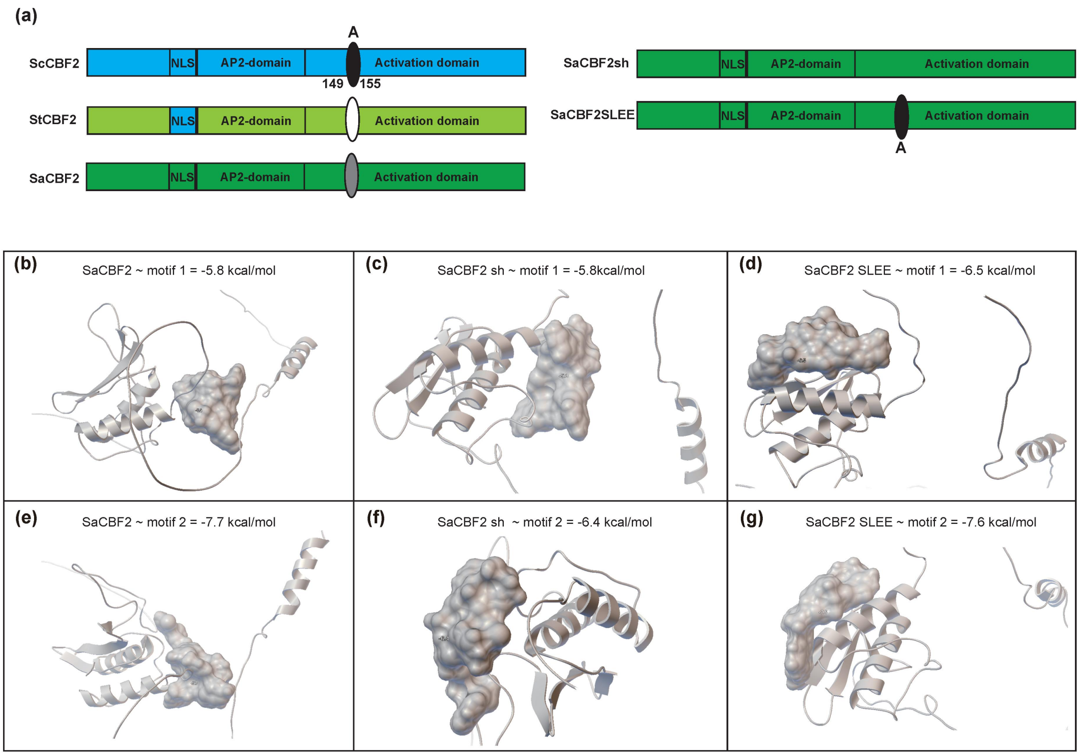

Supplementary Figure S7. Affinity analysis of SaCBF2 and its mutant variants for motif1/2.

(a) Structural representation of SaCBF2 and its mutant proteins. SaCBF2sh represents the deletion of the "S-H" amino acids at site A of the SaCBF2 protein. SaCBF2SLEE represents the substitution of the "S-H" amino acids with "SLEE" amino acids.(b-d) Represent the molecular docking affinity of SaCBF2, SaCBFsh, and SaCBF2SLEE proteins with motif 1, respectively. (e-g) Represent the molecular docking affinity of SaCBF2, SaCBFsh, and SaCBF2SLEE proteins with motif 2, respectively.
