# Supplementary material for: Natural allelic variation in the CBF2 transcription factor is a pivotal factor controlling cold resistance in potato
Source: Plant Physiol. 2025 Oct 15;199(2):kiaf428. doi: 10.1093/plphys/kiaf428 (PMC12559889; doi:10.1093/plphys/kiaf428)
Supplement: kiaf428_Supplementary_Data [file kiaf428_supplementary_data.zip › Supplementary Data 1.docx]

>Pro-ScGolS3-P1

GGTACCCCAAGATTAAGAACGGTGGACGAACAATTTAAAACCTAAAAAGAGAGCTGGACTTTGTGCAAACGCTATCAAGTTTAAAATATGTAACCCTTTCATTTGCCGTGTTCTTGGAAATATTAAGAATGAATAAAATATTTTTGCTTACCGTTTTATGAATAATATATCAGAAGCTTACTTAACTACAACTTATCAATAAGAGAAGTTTAATCATAACTAATTACTCAATTCCTCTATCTAGCAATTTTTTCACAAACTAATTTGTTGCATATAGTTTGGGTGTTAGAAAAAAAAATCTTGTTCAACTTGTGGGTGTCACATACCTTGTAGACAATAACCTAGCAATCAGTAGTCTGGTCCTCTGCCCTACAAAGTAGCTGGATGTGTTTGATCTTAGAGTTATAGAGTTAAATAGACTTCATATTTGTGTGTTATTTTCCATAAAAATCAAATTGTATTAATTTTGTTTGATATTGATTGATATACAGGACGTACGTGTTTGTTTTTTATACATTGTTGATATTATGATATATTGTGCTGTATGAGATTCAAAGGTTTGATCATAATATTAATTAATTTACTTTATCCTAAATTAATAACTCATTAGAAAGCGAGATTAAGGTTTCAACTTTCGTGGTTAATTTATGGAATGAGATAATGGTGGGCCAAATGAAACATTATTCTAGTGTTTTCTACTTTAATTAATTACTGTAGAAAAAGAAAACATTTACTCTATTAATTACTCAAACCACCGCCCAGTTTCCAGAGATTTCTCTTCAATAACAAATAACCCGTCACTATTTGTGATCACAGAAAACAACAAAAAAATCTGAACCGTTGGATCTTATCTGACCACAACACGTACAGTCCGATCCTTGTGGTGCAAAATATAATTTTTAAGATTTCTGGGGCCCAAATGCTCAACTTCAAAGAAACTTACACATATAACGAAACTAAACGACATCGTATTTGCAAAAACACCATCTAATTTATTTTATTTTTTTCATATTTTTAGTTCCCACCCAACGCCTA

>Pro-ScGolS3-P2

CTACAGTCAAAAGGCATGACTCAACTACTCTATTTCATTAGGATAATGTTTTGGTCGGTTTTGTGTGTAACTACAATTATCTCCTTATCTTAACGCTACCATTCAAAATTAACCACACTTGTCAATAAATTCATATTAACTTTCACACACAAGTGTTAACTAATTCACCCAACTAACATAACTACTACTTACATAATTAAAAAAAAAACAAGTAATTAAATTAGAACAAGTCAGAAAAAGTAGCTCTAAAATATTCATTTTCTAGCAAACGTGGCATATCTTCATCCTTCAATTATGATACGTGGCAATATTTTATGGCTCGTAATATGAAAACACGCTTGACATATGGGCCCAACTTGGGCCCACTTTTAACCTCCCTAACTGTTGTGGGCCCGTTGTCATTGAGATCTGGGACCACAGTGGGCCCTCTTTTAACCTCCATAACTGGTGTGGGGACCAGGCTCATTGAATGAAATTTCTATAGATAGCCCTCACCGAATGGATCACGTTACGTGGTGAGGATTAGCCACGTGGCAGCATATTGATGAGTTTGATCCTTTAATTTTGATCCTAAATAGGTTTTGGTCGGCTACCGTTACAACTTTGTCAACAGTCAAAACCCGCGTTGTTGAGAAAAAGATTTTGTAGTTATTGTACGTAAGGAAAAAAGGAAATATATTCTAGATTATGGTATTTCTTAATCTAGTACTAGTACTCCCTCAGTATCGTTTATTATTTC

>Pro-StGolS3-P1

GGTACCCCAAGATTAAGAACGTTGGACCAACAATTTAAAACCTAAAAAGAGAGCTGGACTTTGTGCAATCGCTATCTAGTTTAAAATATGTAACCCTTTCATTTGCCATGTTCTTGGAAATATTAAGAAAGAATAAAATATTTTTGCTTACCTTTTTATGAATAATATATCAGAAGCTTACTTAACTACAACTTATCAATAAGAGAAGTTTAATCATAATTAATTACTCAACTCCTCTATCTAGCAATTTTTTTTACAAACTAATTTGTTGCATATAGTTTGGGTGTTAGAGAAAAAATATCTTGTTCAACTTGTGGGTGTCACATACCTTGTAGATAACAATCGTAGAAATCAGTAATCTGATCCCCTGCCCTACAAAGTAGCTTGATGTGTTTGATCTTAGAGTTATAGAGTTAAATAGACTTTATATTTGTGTGTTATTTTCCATCAAAATCAAATTGTATTAATTTTGTTTGATATTGATTGATATGCAGGACGTACGTGTTTGTTTTTTATACATTGTTGATATTATGATATATTGTGCTGTATGAGATTCAAAGGTTTGATCATAATATTAATTAATTTACTTTATCCTAAATTAATAACTCATTAGAAAGCGAGATTAAGGTTCCAATTTTCGTGGTTAATTTATGGAATGAGATAATGGTGGGCCAAATGAAACATTATTCTAGTGTTTTCTACTTTAATTAATTACTGTAGAAAAAGAAAACATTTACTCTATTAATTACTCAAATCACCGCCCAGTTTCCAGAGATTTCTCTTCAACAACAAATAACCCGTCACTATTTGTGATCATAGAAAACAACAAAAATATCTGAACCGTTGGATCTTACCTGACCACAACACGTACAGTCCGATCCTTGTGGTGCAAAATATAATTTTTAAGATATCTGGGGCCCAAATGCTCTACTTCAAAGAAACTTACACATATAACGAAACTAAACGACATCGTATTTGCAAAACCACCATGCAATTTTATTTTATTTTTTTCATATTTTTAATTCCCACCCAACGCCTA

>Pro-StGolS3-P2

CTACAGTCAAAAGGCATGACTCAACTACTCTATTTCATTAGGATAATGTTTTGGTCGGTTTTGTGTGTAACTATAATTATCTCCTTATCTTAACGCTACCATTCAAAATTAACCACACTTGTCAATGAATTCATATTAACTTTCACACACAAGTGTTAACTAATTCACCCAACTAACATAACTACTACTTACATAATTAAAAAAAAACAAGTAATTAAATTAGAACAAGTCAGAAAAAGTAGCTCTAAAATATTCATTTTCTAGCAAACGTGGCATATCTTCATCCTTCTATTATGATACGTGGCAATATTTTACGGCTCGTAATATGAAAACACGCTTGACATATGGGCCCAACTTGGGCCCACTTTTAACCTCCCTAACTGTTGTGGGCCCGTTCTCAGTGAGATCTGGGACCACAGTGGGCCCTCTTTTAACCTCCATAACTGCTGTGGGGACCAGGCTCATTGAATGAAATTTCTATAGATAGCCCTCACCGAATGGATCACGTCACGTGGCGAGGATTAGCCACGTGGCAGCATATTGATGAGTCTGATCCTTTAATTTTGATCCTAAATAGGTTTTGGTCGGCAACCGTTACAACTTTGTCAACAGTCAAAACCCGCGTTGTTGAGAAAAAGATTTTGTAGTTATTTTGTACGTAAGGAAAAAAGGAAATATATTCTAGATTATGGTATTTCTTAATCTAGTACTCCCTCAGTATCGTTTATTATTTC

>Pro-ScGolS3-P3

TAAATTATTTTATAGGTTTAAAATAAATTATACTCTATGATATAATAAAGATAAAATGAGTTAATATTTCAAAAGGCCACCCAATTTTGATAATTTATCTAGCAAAGTTATTAAACTTTGTTTTGTATCATTAAATCACTAAACTTAGACTTTTCTATCAATAAAATCACTCAATCAAATTTATCATTAAAAAATTATTGGCAAGACAAAATAAATTATCATTTTTATATCATGTAATTATAAATTCCATAATTAAATAAAATTAAAGAAATCTCATTAAGGACTTTTCACTCCCTCTCTCAAAACTTTTTTAAAAAAATCAATTAAAATGTATGTCCAAACACAATTTTAAATTTCAAAACTTATTTATCAACAATTTTATAAAATAATTTTTTCAAGCTTGAACAAACCTATGTCAACGTTAGCAAACCATGGAATTACGACGGAAAAGTAAGATCTTTACCAGTTATATTTAGCAAGAAATATGTAAATAGATTACACACCAAATGACTTATTAATTAATAAATCCCTACGTGTTCTATTTTGACATTTGTTTATTGCTTTTCTTCCTGTTTTGGATTTAATTTGTTGTTTTGCTTAAATACTTATATTCATTTTTTATTTAGGAGACAAACTAAATATTATGGTATTACAATAATACCAAAATATGTTTCAAGAAAAAATTATTCATTTATTTATTAAATGACGACATAAATTATGCAATAATATCATAAATAATATTTGTTTCGGATTAATTAGTTTGTCCGTACTTTTATTAATAAGCTTTTTTTAATATTATTTATTTGTGGATCCGTGTTTATATGACATAAAAATAATAATTTATTTTGCTATGTCAATATTTTTTAATGATAAATTTGATTGAGTGATTTTATTGATAGAAATGTCTAAGTTTAGTGATTTTATTAATACAAAACAAAGTTCAATGACTTTGCTAGATAAATTCTTAAAGTTGAGTGATCTTATGAGATATTAACTCAGATAAAATAATCAAAAATATTCTTAATTAAAGTTTCTTAAGAGCCTGTAAGAAAATGACACGATACATAATTTGAGACCGTGGAAGTTTCATTAAAATAAAACCCATATGTTTTACAAGGTCTTCAAGTCATGACGTGGTTAATTAAATTTTAATATAACCATTTTCTCAAGTATGTAAGTTCATTCTTTTTCATAGTAATACTTTATAGATCTTGCGTCTATCAATTAAATTGAATAGTTCGAACTTCCAACCAAGCACTGGTCGTAACTTTTTAAATAATTATATTTTCATTATTTTTAATTTTGAAAATAAATATTAATCTTAGTCAAACACTGATTTTTCATAATTTTTTAAAAGTGAAAATATGATTAAATAAGATAATAAAATTATGCAAGACA

>Pro-StGolS3-P3

TAAATTATTTTATAGGTTTAAAATAAATTATACTCTATGATATAATAAAGATAAAATGAGTTAATATTTCAAAAGGCCACCCAATTTTGATAATTTATCTAGCAAAGTTATTAAACTTTGTTTTGTATCATTAAATCACTAAACTTAGACTTTTCTATCAATAAAATCACTCAATCAAATTTATCATTAAAAAATTATTGGCAAGACAAAATAAATTATCATTTTTATATCATGTAATTATAAATTCCATAATTAAATAAAATTAAAGAAATCTCATTAAGGACTTTTCACTCCCTCTCTCAAAACTTTTTTAAAAAAATCAATTAAAATGTATGTCCAAACACAATTTTAAATTTCAAAACTTATTTATCAACAATTTTATAAAATAATTTTTTCAAGCTTGAACAAACCTATGTCAACGTTAGCAAACCATGGAATTACGACGGAAAAGTAAGATCTTTACCAGTTATATTTAGCAAGAAATATGTAAATAGATTACACACCAAATGACTTATTAATTAATAAATCCCTACGTGTTCTATTTTGACATTTGTTTATTGCTTTTCTTCCTGTTTTGGATTTAATTTGTTGTTTTGCTTAAATACTTATATTCATTTTTTATTTAGGAGACAAACTAAATATTATGGTATTACAATAATACCAAAATATGTTTCAAGAAAAAATTATTCATTTATTTATTAAATGACGACATAAATTATGCAATAATATCATAAATAATATTTGTTTCGGATTAATTAGTTTGTCCGTACTTTTATTAATAAGCTTTTTTTAATATTATTTATTTGTGGATCCGTGTTTATATGACATAAAAATAATAATTTATTTTGCTATGTCAATATTTTTTAATGATAAATTTGATTGAGTGATTTTATTGATAGAAATGTCTAAGTTTAGTGATTTTATTAATACAAAACAAAGTTCAATGACTTTGCTAGATAAATTCTTAAAGTTGAGTGATCTTATGAGATATTAACTCAGATAAAATAATCAAAAATATTCTTAATTAAAGTTTCTTAAGAGCCTGTAAGAAAATGACACGATACATAATTTGAGACCGTGGAAGTTTCATTAAAATAAAACCCATATGTTTTACAAGGTCTTCAAGTCATGACGTGGTTAATTAAATTTTAATATAACCATTTTCTCAAGTATGTAAGTTCATTCTTTTTCATAGTAATACTTTATAGATCTTGCGTCTATCAATTAAATTGAATAGTTCGAACTTCCAACCAAGCACTGGTCGTAACTTTTTAAATAATTATATTTTCATTATTTTTAATTTTGAAAATAAATATTAATCTTAGTCAAACACTGATTTTTCATAATTTTTTAAAAGTGAAAATATGATTAAATAAGATAATAAAATTATGCAAGACA
